# Pantranscriptome combined with phenotypic quantification reveals germplasm kinship and regulation network of bract color variation in *Bougainvillea*

**DOI:** 10.3389/fpls.2022.1018846

**Published:** 2022-11-17

**Authors:** Huaxing Huang, Hongli Ji, Song Ju, Wei Lin, Jing Li, Xuanrui Lv, Lixian Lin, Lijin Guo, Dongliang Qiu, Jianyong Yan, Xiaokai Ma

**Affiliations:** ^1^ Center for Genomics and Biotechnology, School of Future Technology, Haixia Institute of Science and Technology, Fujian Agriculture and Forestry University, Fuzhou, China; ^2^ Yuanshan Institute of Bougainvillea in Longhai, Zhangzhou, China; ^3^ Vegetable and Flower Institute, Jiangxi Academy of Agricultural Sciences, Nanchang, China; ^4^ College of Forestry, Fujian Agriculture and Forestry University, Fuzhou, China; ^5^ College of Horticulture, Fujian Agriculture and Forestry University, Fuzhou, China; ^6^ College of Life Sciences, Fujian Agriculture and Forestry University, Fuzhou, China; ^7^ International Magnesium Institute, School of Future Technology, Haixia Institute of Science and Technology, Fujian Agriculture and Forestry University, Fuzhou, China; ^8^ Key Laboratory of Orchid Conservation and Utilization of National Forestry and Grassland Administration at College of Landscape Architecture, Fujian Agriculture and Forestry University, Fuzhou, China

**Keywords:** pan-transcriptome, *Bougainvillea* bracts, color variation, co-expression network, gene regulation

## Abstract

Bracts are the metamorphic non-flower organ in angiosperm plants. The variation of the color and shape of bracts was found to be neo-functionalized (i.e., similar to petals), garnering research interest as a pollinator attractor. *Bougainvillea* is known for its specialized, large, and colorful bracts, which contrast with its tiny colorless flowers. As a plant whose bracts vary greatly in terms of coloration, the molecular mechanisms for *Bougainvillea* bract coloration and polychroism are largely unknown. The lack of genomic information for *Bougainvillea* largely hinders studies into the evolution and genetic basis of bract color variation. In this study, a pan-transcriptome of bracts obtained from 18 *Bougainvillea glabra* accessions was employed to investigate the global population-level germplasm kinship and the gene regulation network for bract color variation. Our results showed that the bracts of *B. glabra* accessions have largely differentiated International Commission on Illumination (CIE) L-a-b values. Moreover, germplasm kinship detected using principal component analysis, phylogeny, and admixture analysis showed three optimal subgroups, two of them distinctly clustered, which were not directly correlated with bract color variation at the population level. Differentially expressed genes (DEGs) between accessions of high vs. low L-a-b values revealed several considerable upregulated genes related to bract color L-a-b variation. A weighted gene co-expression network was constructed, and eight co-expressed regulation modules were identified that were highly correlated with variation in bract CIE L-a-b color values. Several candidate DEGs and co-expressed hub genes (e.g., *GERD*, *SGR*, *ABCA3*, *GST*, *CYP76AD1*, *CYP76C*, and *JAZ*) that were tightly associated with bract color variation were eventually determined responsible for L-a-b colorations, which might be the core regulation factors contributing to the *B. glabra* bract color variation. This study provides valuable insights into the research on germplasm kinship, population-level pan-transcriptome expression profiles, and the molecular basis of color variation of key innovative bracts in horticultural *Bougainvillea*.

## Introduction

Even though the perianth (e.g., petals) has always been responsible for attracting pollinators and protecting reproductive organs (Pelaz et al., 2001; Ronse De Craene, 2007; Ma et al., 2016), the variation in color and shape makes them the main ornamental organs of horticultural plants (Colinas et al., 2014; Roy et al., 2015; Grueber, 2019). Compared with flower organs, bracts (usually metamorphosed leaves), which are non-floral organs, have received less attention. The eco-physiological function of bracts is not narrow and is thought to be neo-functionalized like the petals. The function of bracts is not only related to long-distance visual and reward signals but also for protecting the flower organs (Borges et al., 2003; Sun et al., 2008; Pélabon et al., 2012; Song et al., 2013; Chen et al., 2015). It has been reported that bracts act as signal amplifiers in hummingbird vision for flower pollination (Bergamo et al., 2019). In low-density butterfly-pollinated plants, in the absence of flowers, the white ultraviolet (UV)-absorbing bracts still attract butterflies to the plant (Borges et al., 2003). The bracts below the flowers of the epiphytic *Neoregelia punctatissima* in tropical rain forests can keep water stagnant and form a mutually beneficial symbiosis with *Utricularia vulgaris* (Benzing, 1989; Armbruster, 1997; Studnicka, 2009). It has been reported that the huge bracts of the Himalayan special giant herb, *Rheum nobile*, have multiple functions that not only provide heat within the flower and reduce UV radiation reaching the fruit but also could prevent pollen grains from being washed away by rain and increase the number of flower visitors (Song et al., 2013). The bracts of some tropical plants gradually changed from small to large, nocturnal open to closed, monochromatic to multicolored and evolved special functions to defend against natural enemies and attract more pollinators. In general, a bract is functionally similar to a petal and is an important organ within the context of plant reproductive strategies (Fenster et al., 2004).

In ornamental applications, many plants are exploited for their bright bract coloration, variable morphology, such as *Bougainvillea* spp., *Davidia involucrata*, *Anthurium andraeanum*, *Zantedeschia aethiopica*, *N. punctatissima*, *Euphorbia pulcherrima*, and *Euphorbia milii*. Several studies focus on the mechanism of development, color formation, and surface structure of bracts, among which some are based on the metabolomics-associated transcriptome (Li et al., 2016; Li et al., 2018; Nitarska et al., 2018). Moreover, genes related to bract color regulation have been identified from some of these plant species (Collette et al., 2004; Gopaulchan et al., 2014; Wei et al., 2016; Nitarska et al., 2021). Vilperte et al. (2021) indicated that the *GST* gene with a high mutation rate alters the changeable bract coloration in *E. pulcherrima*. Feng et al. (2012) found that B-class gene family may have contributed to the origin of bract in *Cornus florida*. Zhang et al. (2022) revealed that ABE class genes in the ABCDE model of floral development were highly expressed in *Bougainvillea glabra*.

The colorful bracts of *Bougainvillea* have been reported to attract moths and hummingbirds to assist in flower pollination in the tropical areas of Brazil (Bergamo et al., 2019). Moreover, the bract of *Bougainvillea* is one of the most variable plant ornamental organs regarding its color and shape. *B. glabra* is a globally popular horticultural plant that is widely planted in various gardens and landscapes due to its colorful bracts and free flowering habit (Roy et al., 2015; Saleem et al., 2021). *Bougainvillea* is a betalain biosynthesis plant; however, its bract coloration is driven by the difference in the content and ratio of betacyanins and betaxanthins according to a recent study (Wu et al., 2022). Although there are a total of 18 species in the genus *Bougainvillea*, only four species including *B. glabra* have large, colorful, and ornamental bracts (Kobayashi et al., 2007; Saleem et al., 2020). With nearly 200 years of cultivation, there are approximately 600 cultivars globally (Roy et al., 2015). Some of their bracts are in a wide range of colors, *viz.*, white, yellow, pink, red, mauve, bicolored, and multicolored. *B. glabra* has an easy-bloom habit, fewer diseases and pests, and strong growth vigor and is used extensively in bonsai, gardens, and urban landscaping. Portable potted *B. glabra* plants of unique shape and size are a million-dollar business when nurseries are concerned (Roy et al., 2015). *B. glabra* not only is popular in ornamental applications but also recently has been used in biochemistry and the biosynthesis of compounds related to nutrition, medicine, and photovoltaic areas (Hernandez-Martinez et al., 2011; Abarca-Vargas et al., 2016; Abdel-Salam et al., 2017; Ahmar Rauf et al., 2019; Ashmawy et al., 2020; Saleem et al., 2020).

Even though there are lots of applications for *Bougainvillea* bracts, there is still a gap in the study for the molecular basis of the extensive variation of bract coloration in *B. glabra*. The function of bracts within the context of plant–pollinator interactions, adaptive evolution, and genetic basis of reproductive innovations has been seldomly investigated. Despite the plant having large colorful bracts, there is no reference genome available to ease the genome-wide association studies. To date, the molecular regulation mechanism of color variation in bracts of *B. glabra* at a large population level remains unexploited. There are some transcriptomic studies, but they are not sufficient to explain the large population-level phenotypic variations (Zhang et al., 2022). In this study, a pan-transcriptome assembly was employed based on transcriptome sequence data of 18 *B. glabra* accessions. The variation of bract coloration was quantified by measuring the International Commission on Illumination (CIE) L-a-b values using a spectrometer. The germplasm kinship within these accessions was detected at the global population level by principal component analysis (PCA), a phylogenetic tree, and admixture analysis. Moreover, an analysis of differentially expressed genes (DEGs) related to the high vs. low L-a-b value of bract coloration was conducted. In addition, the weighted gene co-expression network analysis (WGCNA) was performed between pan-transcriptome expression profiles and bract color variation of CIE L-a-b data to construct the essential regulation networks and identify the core regulation factors for *B. glabra* bract coloration. Finally, the pan-transcriptome profiles linked to *B. glabra* population-level color variation and associated regulation mechanisms were discussed.

## Materials and methods

### Sample collection

Bract samples used in this study were obtained from cuttings (3–5 years old) of *B. glabra* cultivars or accessions cultivated in the nursery of the Institute of Bougainvillea in Yuanshan, Zhangzhou, China. For each examined sample, mature bracts on several branches from a single accession were used. The information on the sampled accessions was listed in **Supplementary Table S1**.

### Bract color measurements

In order to quantify the variation of bract coloration, the CIE L-a-b value (i.e., L, representing the brightness dimension; a, value representing the red and green dimension; b, representing the yellow and blue dimension) of standard color space model was used to measure the bract surface color using the AvaSpec-ULS2048CL-EVO high-speed CMOS spectrometer [Avantes (Shanghai, China) Co., Ltd.] (Ogutcen et al., 2020). The measurement of L-a-b color values for each accession was repeated four times. The average value (AL, Aa, Ab) of each L-a-b value was calculated as color trait data.

### RNA sequencing and transcriptome assembly data source

The RNA sequencing (RNA-seq) data and *de novo* transcriptome assembly were downloaded from the Genome Sequence Archive (GSA), National Genomics Data Center (NGDC), under Bioproject number PRJCA011746 (Supplementary Materials).

### Read mapping and variant calling

The transcriptome reads of the 18 *B. glabra* accessions were mapped onto the *de novo* assembled pan-transcriptome assembled transcripts using Bowtie2 (Langmead and Salzberg, 2012). The variants [including single-nucleotide polymorphisms (SNPs) and Indels] were called from mapped BAM files using the GATK pipeline with HaplotypeCaller model (McKenna et al., 2010). The variants were filtered using the following parameters: 2 < depth (DP) < 100, minQ >20, maximum missing rate <40%, and minor allele frequency (MAF) >2%. The filtered set of variants was then used for the downstream population genomic analysis.

### Population genomic analysis

A maximum likelihood (ML) tree of the 18 *B. glabra* accessions was constructed with filtered variant sets using the software IQ-TREE 2.1.3 (Lam-Tung et al., 2015). Iterative tree construction was performed using the best model, PMB+F+R3 protein model, according to the Bayesian Information Criterion and an ultrafast bootstrap approximation algorithm. The final ML tree was visualized using the software iTOL v4 (Letunic and Bork, 2019).

To further analyze the genetic relationship among the 18 accessions, the population admixture was constructed using admixture v1.3.0 (Alexander et al., 2009). The optimal number of clusters (K) was identified based on the cross-validation (CV) error value, which was tested from K = 1 to 10. After choosing the optimal K value, the admixture matrix was visualized using iTOL v4.

The genetic relationship matrix among the 18 accessions was calculated using the software GCTA (Yang et al., 2011). The eigenvalues and eigenvectors were generated for PCA. The top 2 principal components were used to generate PCA scatter plot by ggplot2 in R (RAM and ZJ, 2019).

### Pan-transcriptome expression analysis

The expression levels of unigenes for bracts among the 18 accessions were quantified using the software RSEM (Dewey and Li, 2011) using BAM files generated from read mapping and normalized to the transcript per million (TPM) matrices. The statistical distribution of expression was constructed in box plot. The pheatmap package in R (Kolde and Kolde, 2018) was used to construct a heatmap with clustering tree for the correlation of expression levels among the 18 accessions. The PCA dimensionality reduction analysis was also performed on the expression levels of bracts to identify the clusters of the 18 accessions with high expression similarity.

### Differentially expressed gene analysis

In DEG analysis, expression data of three accessions with the highest AL values vs. three accessions with the lowest AL values were used for comparison in DESeq2 (Love et al., 2014) with multiple test correction method “BH.” Significantly DEGs with *P*-adjust values <0.05 and two times up/down fold changes were screened. Expression data of accessions with the highest and the lowest Aa and Ab values were also processed in a similar way.

### Weighted gene correlation network analysis and hub gene identification

To determine the correlation between bract phenotypical color variation and their gene expression levels, a gene expression network based on color variant trait data and gene TPM expression data of bracts from the 18 accessions was constructed using the WGCNA pipeline (Langfelder and Horvath, 2008). After the invalid insignificant expressed genes were filtered out from the TPM data, soft threshold of unsigned network was calculated to match the subsequent correlation analysis. Module detection was performed by Topological Overlap Matrix (TOM)-based similarity measurements, and the minimum number of genes per module was set at 50 genes. The module genes were then divided, while the similar modules were merged. For the module–trait correlation analysis, the eigengene value was calculated for each module to test the association with each average L-a-b color trait. The results of module–trait correlation were then constructed into heatmap to pick essential modules for subsequent analysis.

The module genes with high correlation with color trait data were exported to calculate the weight nodes and screen the hub genes in the cytoHubba plug-in of Cytoscape software (Shannon et al., 2003). Maximal Clique Centrality (MCC) algorithm was used to calculate the top 50 weighted genes in picked module and saved to map former annotation. After essential modules were selected and hub genes were calculated, a co-expression network was constructed using software Gephi (Bastian, 2009). We uploaded each picked module gene and hub gene with their annotation. Edge degree and module classification was calculated in Gephi, and small nodes without annotation and high edge degree were filtered. Finally, the co-expression network for each module was constructed.

### Gene Ontology and Kyoto Encyclopedia of Genes and Genomes enrichment analysis

The selected genes from the DEG analysis and the genes in the essential modules from WGCNA were enriched based on the Gene Ontology (GO) and Kyoto Encyclopedia of Genes and Genomes (KEGG) database using clusterProfiler package in R (Yu et al., 2012). The top 20 terms of GO and KEGG enrichment were visualized in dotplot. The significance levels of GO and KEGG enrichment were tested with standard of *P* < 0.05.

### Expression patterns of candidate differentially expressed genes and hub genes

The candidate genes from DEG analysis and the hub genes in the top WGCNA modules that correlated with L-a-b values were selected for expression pattern analysis. The expression patterns of selected genes were visualized using pheatmap package in R (Kolde and Kolde, 2018) according to the order of each bract L-a-b value from high to low among the 18 *B. glabra* accessions.

### RT-qPCR expression validation

Real-time qPCR (RT-qPCR) was designed to verify the expression trend of candidate genes, and they were compared with transcriptome expression data (Bustin et al., 2009). Candidate genes screened out from DEGs and WGCNA were subjected to RT-qPCR experiment. Total RNA from bract samples with high, medium, and low L-a-b values was extracted for RT-qPCR using a Tiangen polysaccharide and polyphenol plant total RNA extraction kit [Tiangen Biotech (Beijing) Co., Ltd.]. The primers were designed by the Primer Premier 5.0 (Lalitha, 2000) (**Supplementary Table S2**). cDNA was synthesized using a TaKaRa PrimeScript RT Reagent Kit with a gDNA Eraser (Perfect Real Time) kit. RT-qPCR was performed on the CFX 96™ Real-Time PCR Detection System. The relative expression data were quantified by the 2^−△△CT^ method and normalized by 18S rRNA (Schmittgen and Livak, 2008). The reaction mixture (20 μl) contained 10 μl of TB Green Premix Ex Taq II (Tli RNaseH Plus) (2×), 0.5 μl of PCR Forward Primer (10 μM), 0.5 μl of PCR Reverse Primer (10 μM), 1 μl of DNA template, and 8 μl of ddH_2_O. Subsequently, the PCR programs were conducted with a pre-denaturation at 95°C for 3 min; 40 cycles of 95°C for 10 s, 60°C for 30 s; and a final 65°C–95°C slow increase for melting curve analysis. Finally, *T*-tests were performed to detect the significance between differential expressions between samples.

## Results

### Phenotypic quantification of bract color variation in *Bougainvillea glabra*

**Figure 1:**
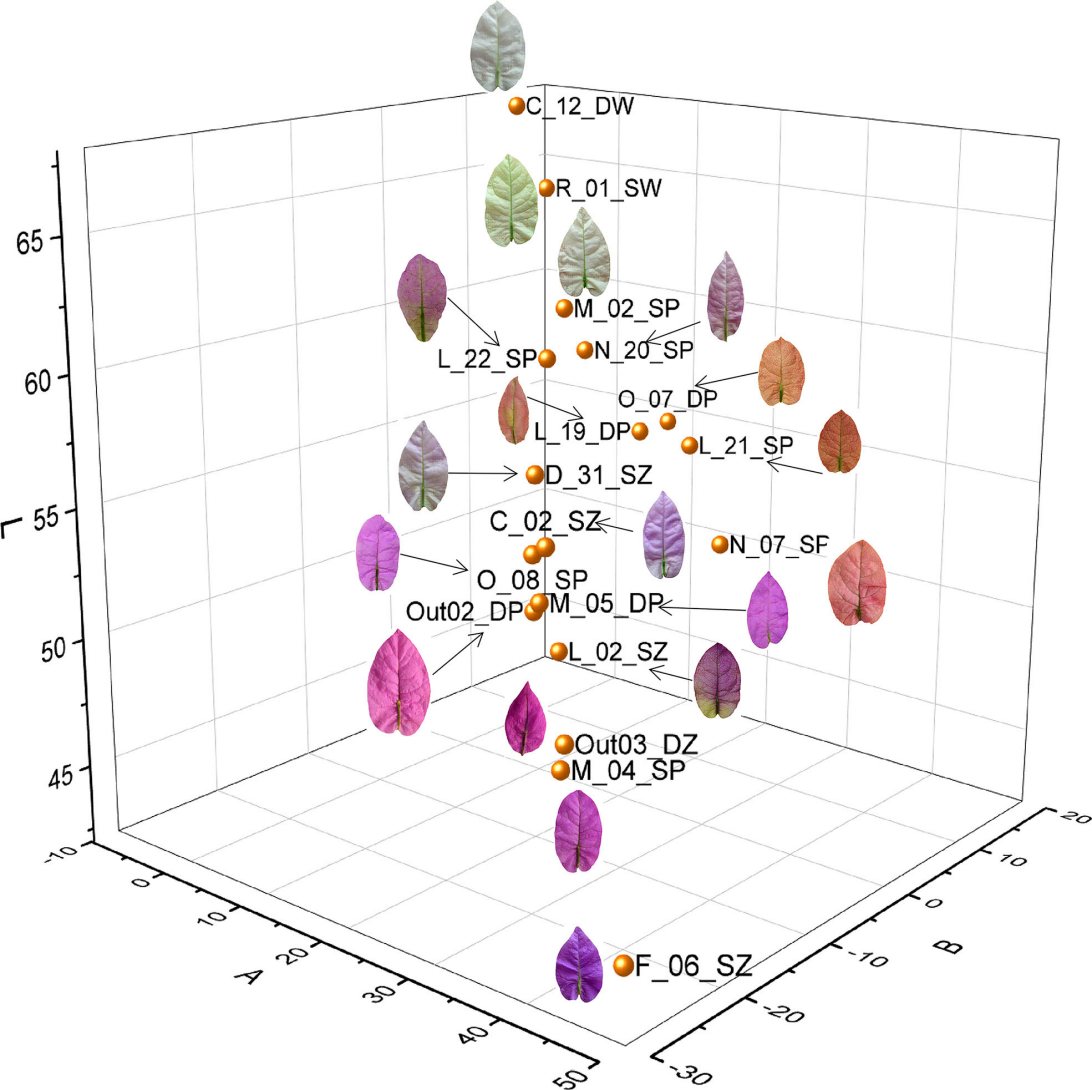
Cluster of bract color variations based on L-a-b value of the 18 *Bougainvillea glabra* accessions. There were three dimensions belonging to L, a, and b values, with red-green dimension (Aa), yellow-blue dimension (Ab), and brightness dimension (AL). Each point represents each accession.

The color variation of bract surface among the 18 accessions was quantified and represented by L-a-b values (**Supplementary Tables S1, S3**) and visualized as a three-dimensional (3D) scatter plot (**Figure 1**). In each dimension of L-a-b, the color data presented a linear variation. On the red-green dimension (trait Aa), F_06_SZ had the highest red value with 44.81 and C_12_DW had the highest green value with -1.983. On the yellow-blue dimension (trait Ab), O_07_DP had the highest yellow value with 15.38 and F_06_SZ had the highest blue value with -24.88. On the brightness dimension (trait AL), C_12_DW had the brightest value with 67.90 and F_06_SZ had the darkest value with 43.04. According to the measurement result, as |Aa+| (red) and |Ab-| (blue) values increased, the bract color tended to change from light purple to dark purple. When |Aa+| and |Ab+| (yellow) values increased, the bract color tended to change from pink to orange. When |Aa-| (green) and |AL+| (brightness) values increased, the bract color changed from white to bright green, giving it a leaflike appearance. Appropriately, F_06_SZ is the darkest and deepest purple accession and C_12_DW is the brightest whitest accession without any red pigmentation. Accessions O_07_DP and L_21_SP had top |Ab+| value, and the |Aa| value was not less than 10 that gave them orange coloration with a little red blended pigmentation.

### Population structure of *Bougainvillea glabra* accessions

**Figure 2:**
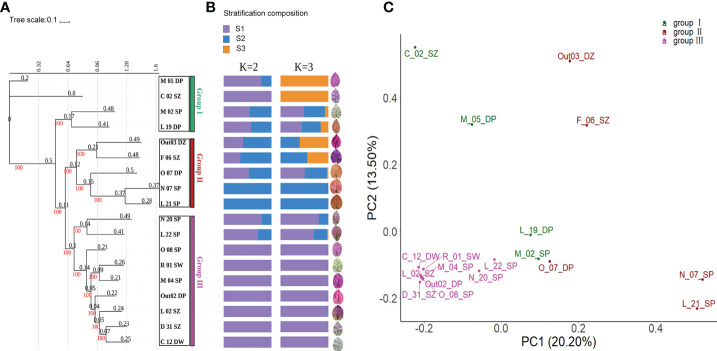
Germplasm kinship of the 18 *Bougainvillea glabra* accessions. **(A)** Maximum likelihood (ML) phylogenetic tree. The red numbers represent the bootstrap supporting values that were all 100%. The black numbers represent the length of each tree branch. Three colorful frames represent three clusters (Groups I, II, and III) of the 18 accessions on the tree. **(B)** Population structure. K = 2 and K = 3 show the two optimal inferred ancestral stratifications. **(C)** Principal component analysis (PCA) of *B*. *glabra* accessions. The top 2 principal components with 20.20% (PC1) and 13.50% (PC2) variation contributions. The three clustered groups (Groups I, II, and III) were consistent with the clusters on the ML tree.

The phylogenetic tree clustered the 18 accessions into three major subgroups: Group I, Group II, and Group III (**Figure 2A**). Group I (C_02_SZ, L_19_DP, M_02_SP, and M_05_DP) clustered dispersedly. The accessions in Group I have a similar bract shape, which was narrowly elongated and sharply pointed at the front, while their bract color varied from white to pink-purple. Group II (Out_03_DZ, F_06_SZ, L_21_SP, N_07_SP, and O_07_DP) clustered apparently. Group II accessions also have a similar bract shape, which was mostly bigger and round overall, slowly pointed at the front, and slowly convergent at the base. Group III (C_12_DW, D_31_SZ, L_02_SZ, L_22_SP, M_04_SP, N_20_SP, O_08_SP, Out02_DP, and R_01_SW) clustered densely. The accessions in Group III mostly have round overall bracts with color varying from pink-purple to white.

Admixture analysis showed that the optimal clusters of the 18 accessions are K = 2 and K = 3 as revealed by the lowest CV error (**Figure 2B**). When K = 2, Group III of the phylogenetic tree mostly consisted of S1 stratification in admixture groups. Group I and Group II of the tree consisted of two stratifications, S1 and S2. When K = 3, most of the accessions in Group III only consisted of S1 stratification. Group I and Group II of the tree consisted of one, two, or three stratifications. The accessions N_07_SP and L_21_SP of Group II consisted of only S2 stratification, while C_02_SZ and M_05_DP of Group I consisted of only S3 stratification.

The clustering results of PCA were consistent with that of the phylogenetic tree and population structure. According to the PCA scatter plot of the 18 accessions (**Figure 2C**), the first two principal components in the PCA explained 20.20% and 13.50% of variation, respectively, with a combined 33.70% of the total variation.

### Pan-transcriptome expression profiles of *Bougainvillea glabra* bracts

**Figure 3:**
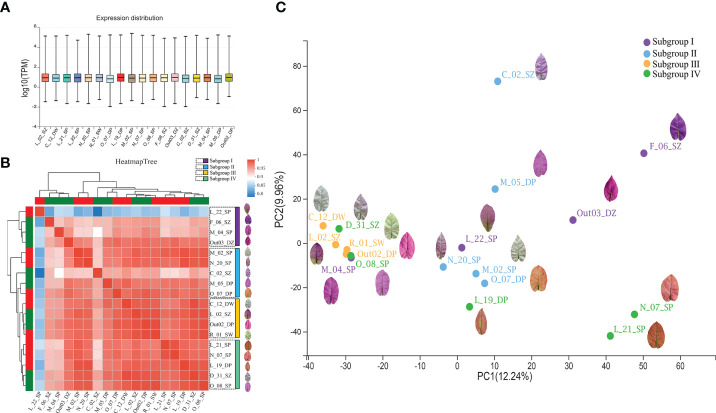
Pan-transcriptome expression profiles of the 18 *Bougainvillea glabra* accessions. **(A)** The distribution of expression levels of the 18 accessions. The x axis represents each accession, and the y axis represents the log_10_(TPM) value. **(B)** The correlation of expression levels among the 18 accessions based on expression level (TPM values). Heatmap tree represents the magnitude of the correlation coefficient among accessions, which was clustered into four subgroups (Subgroups I, II, III, and IV). **(C)** Principal component analysis (PCA) of the 18 accessions based on expression level (TPM values). The top 2 principal components with 12.24% (PC1) and 9.96% (PC2) variation contributions. The four clustered groups (Subgroups I, II, III, and IV) were consistent with the clusters on the heatmap tree.

To detect the expression variation and their relationships among *B. glabra* accessions, we analyzed the expression distribution and correlation of all unigenes based on the pan-transcriptome assembly of the 18 accessions. The expression distribution was revealed by the log_10_(TPM) values in box plot (**Figure 3A**). The expression TPM level of each accession varied between -2 and 6. A total of 23,857 unigenes were expressed in all accessions, 11,100 unigenes were expressed in the most orange bract accessions O_07_ DP and L_21_SP, and 9,188 unigenes were expressed only in C_12_DW, which has the whitest bracts.

The correlation analysis of expression level showed that the 18 accessions were clustered into four subgroups (**Figure 3B**). Subgroup I contained accessions F_06_SZ, M_04_SP, Out_03_DZ, and L_22_SP, among which three of them had the deepest purple bracts. Subgroup II contained accessions M_02_SP, N_20_SP, C_02_SZ, M_05_DP, and O_07_DP, among which three of them had light purple bracts. Subgroup III consisted of accessions C_12_DW, L_02_SZ, Out02_DP, and R_01_SW, which had two white bract accessions. Subgroup IV contained accessions L_21_SP, N_07_SP, L_19_DP, D_31_SZ, and O_08_SP, among which three of them had orange bracts.

According to the subgroup classification in the correlation analysis, the PCA clustering of expression levels among the 18 accessions (**Figure 3C**) showed that Subgroup I separated dispersedly and most accessions are purple bracts. Several accessions of Subgroup II clustered together on PC1 dimension but dispersedly along PC2 dimension whose bract color varied from purple to orange. Subgroup III clustered densely that were mostly purple bract and white bract accessions. Subgroup IV contained three accessions with orange bract relatively clustered together on PC2 but dispersedly on PC1 dimension.

### Differentially expressed gene analysis of bract color traits

**Figure 4:**
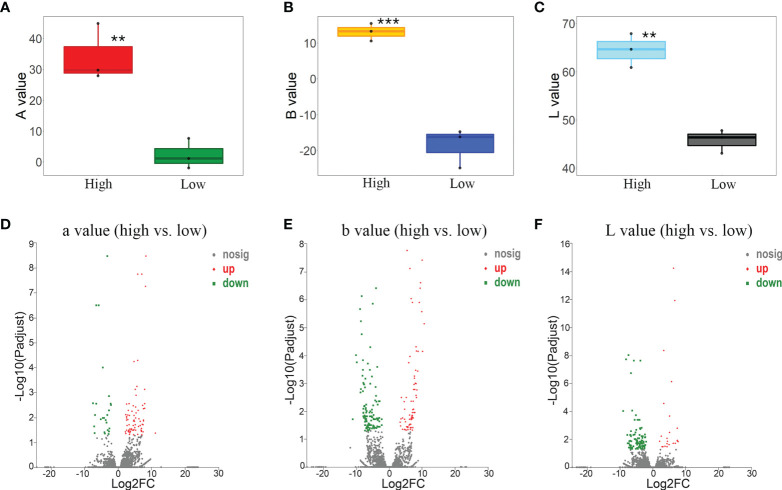
Differentially expressed gene (DEG) analysis based on the pairwise contrast comparisons of bract color L-a-b values. **(A)** Box plot of a value between accessions of high (F_06_SZ, M_04_SP, and Out_03_DZ) vs. low (N_20_SP, C_12_DW, and R_01_SW) values. **(B)** Box plot of b value between accessions of high (O_07_DP, L_21_SP, and L_19_DP) vs. low (F_06_SZ, M_04_SP, and Out_03_DZ) values. **(C)** Box plot of L value between accessions of high (C_12_DW, R_01_SW, and M_02_SP) vs. low (F_06_SZ, M_04_SP, and Out_03_DZ) values. Volcano plot of DEGs for a **(D)**, b **(E)**, and L **(F)** value of each comparison between accessions with high vs. low values. Asterisks represent the significance of the *T*-test for each comparison as indicated by **P < 0.01 and ***P < 0.001.

To screen the gene response to the bract color variation, accessions with the highest and lowest L-a-b values were selected to conduct the DEG analysis. By comparing the gene expressions of the three accessions (N_20_SP, C_12_DW, and R_01_SW) with the lowest Aa values and the three accessions (F_06_SZ, M_04_SP, and Out_03_DZ) with the highest Aa values, 101 significantly upregulated/downregulated genes (76 upregulated/25 downregulated) were identified (**Figures 4A, D**; **Supplementary Table S4**). These genes were mostly involved in the extracellular region (gene number/*P* value, 8/0.00), cell wall (6/0.00), external encapsulating structure (6/0.00) according to GO enrichment (**Supplementary Figure S1A** left) and were enriched in plant–pathogen interaction (3/0.02), cyanoamino acid metabolism (2/0.00), and pentose and glucuronate interconversions (2/0.01) according to KEGG enrichment (**Supplementary Figure S1A** right).

By comparing the gene expressions of the three accessions (F_06_SZ, M_04_SP, and Out_03_DZ) with the lowest Ab values and the three accessions (O_07_DP, L_21_SP, and L_19_DP) with the highest Ab values, 196 significantly upregulated/downregulated genes (75 upregulated/121 downregulated) were identified (**Figures 4B, E**; **Supplementary Table S4**). These genes were mostly involved in the extracellular region (6/0.01), monooxygenase activity (5/0.00), and cell wall organization or biogenesis (4/0.01) according to GO enrichment (**Supplementary Figure S1B** left) and were enriched in plant–pathogen interaction (5/0.00) and phenylpropanoid biosynthesis (4/0.00) according to KEGG enrichment (**Supplementary Figure S1B** right).

According to the comparisons of the gene expressions for the three accessions (F_06_SZ, M_04_SP, and Out_03_DZ) with the lowest AL value and the three accessions (C_12_DW, R_01_SW, and M_02_SP) with the highest L value, 119 significantly upregulated/downregulated genes (23 upregulated/96 downregulated) were identified (**Figures 4C, F**; **Supplementary Table S4**). These genes were mostly involved in the extracellular region (11/0.00), catabolic process (8/0.01), and defense response (6/0.00) according to GO enrichment (**Supplementary Figure S1C** left) and were enriched in sesquiterpenoid and triterpenoid biosynthesis (2/0.01) and pentose and glucuronate interconversions (2/0.02) according to KEGG enrichment (**Supplementary Figure S1C** right).

### Weighted gene co-expression network analysis and hub gene identification

**Figure 5:**
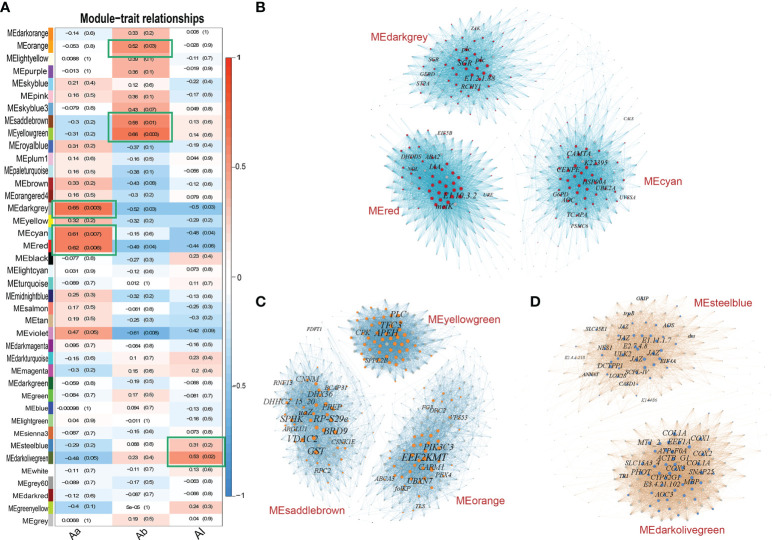
Relationships between WGCNA modules and bract color traits and co-expression networks of top modules. **(A)** Module–trait correlations show 40 modules were clustered in the heatmap. The x axis represents the L-a-b value; the y axis represents clustered modules. The heatmap shows the Pearson correlation coefficient (PCC) with *P* values. The top WGCNA modules (green frames) were selected by criteria of PCC value >60% for Aa value, PCC value >50% for Ab value, and PCC value >30% for AL value. **(B)** Networks of the top 3 modules MEdarkgrey, MEred, and MEcyan correlated with Aa value. A total of 96 annotated hub genes are shown by colored nodes in these modules. **(C)** Networks of the top 3 modules MEyellowgreen, MEsaddlebrown, and MElightyellow correlated with Ab value. A total of 98 annotated hub genes are shown by colored nodes in these modules. **(D)** Networks of the top 2 modules MEdarkolivegreen and MEsteelblue correlated with AL value. A total of 62 annotated hub genes are shown by colored nodes in these modules. In each network, the node represents the gene, and the edge represents the connection between nodes. The size of node and word represents the weight degree (number of nodes it connected).

The L-a-b values as the trait data combined with a total of 19,484 gene expression data of bracts for the 18 accessions were subjected to WGCNA. We picked the soft threshold (power) at 7, which passed the scale independence and mean connectivity standard for the network analysis. Gene expression was detected as an outlier based on the “ward.D2” module with all accessions passed. A total of 40 modules were constructed in the WGCNA (**Figure 5A**). Modules MEdarkgrey, MEred, and MEcyan had more than 60% correlation (Pearson correlation coefficient) with trait Aa but low to -1% for traits Ab and AL. Modules MEyellowgreen, MEsaddlebrown, and MEorange had more than 50% correlation with trait Ab. Only module MEdarkolivegreen had more than 50% correlation with trait AL. The genes in the modules that had more than 60% correlation with bract color Aa value were constructed into a co-expression network by Gephi (**Figure 5B**) and calculated by the cytoHubba plug-in in Cytoscape based on the MCC algorithm.

The top 50 genes in the co-expression network were selected as the hub genes of each module according to the connect weight values calculated by Cytoscape. A total of 96 notable annotated hub genes from co-expression networks of the top 3 modules were screened (**Figure 5B**; **Supplementary Table S5**). For trait Ab, the genes in the modules that had more than 50% correlation with bract color Ab value were constructed into the co-expression network. A total of 98 annotated hub genes from co-expression networks of the top 3 modules were selected (**Figure 5C**; **Supplementary Table S5**). For trait AL, the genes in the modules that had more than 30% correlation with L value were constructed into the network. A total of 62 annotated hub genes from co-expression networks of the top 2 modules were selected (**Figure 5D**; **Supplementary Table S5**).

### Gene Ontology and Kyoto Encyclopedia of Genes and Genomes enrichment of genes in Weighted Gene Correlation Network Analysis (WGCNA) modules

**Figure 6:**
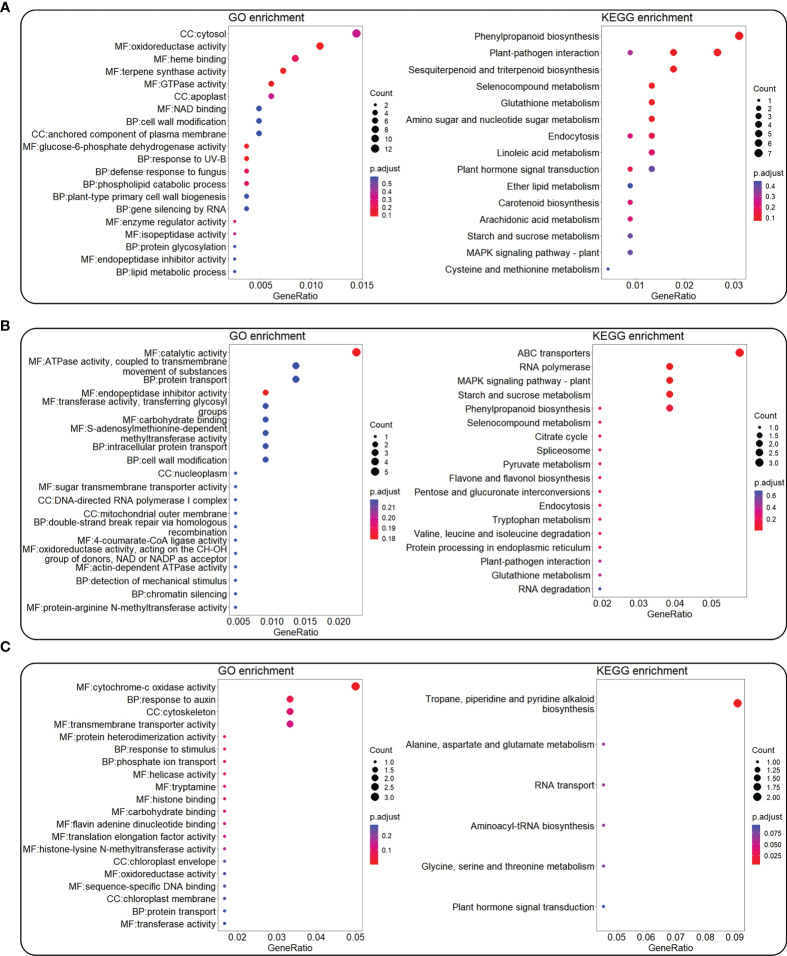
GO and KEGG enrichment for genes in the top WGCNA modules correlated with color L-a-b values. The top WGCNA modules were selected by criteria of Pearson correlation coefficient value >50% and *P* < 0.05 from **Figure 5**. GO (left) and KEGG (right) enrichment for genes in top modules co-related with Aa **(A)**, Ab **(B)**, and AL **(C)** values. The color bar means the *P*-adjust value, while the circle shows the number of enriched genes.

Genes in the modules that had more than 50% Pearson correlation coefficient with each color trait value and *P* value <0.05 were extracted for GO and KEGG enrichment (**Figure 6**). For trait Aa, the genes in modules MEdarkgrey (Pearson correlation coefficient/*P* value, 0.65/0.003), MEcyan (0.61/0.007), and MEred (0.62/0.006) were selected for enrichment analysis. GO enrichment indicated that 1,621 genes in these top modules were mainly involved in oxidoreductase activity (gene number/*P* value, 9/0.00), terpene synthase activity (6/0.00), GTPase activity (5/0.00), and response to UV-B (3/0.00) (**Figure 6A** left). KEGG enrichment indicated that 1,621 genes were mainly enriched in phenylpropanoid biosynthesis (7/0.01), plant–pathogen interaction (6/0.01), and sesquiterpenoid and triterpenoid biosynthesis (4/0.00) (**Figure 6A** right).

For trait Ab, genes in the modules MEorange (0.52/0.03), MEsaddlebrown (0.58/0.01), and MEyellowgreen (0.66/0.003) were selected for enrichment analysis. GO enrichment indicated that 473 genes in these top modules were mainly involved in catalytic activity (5/0.00), ATPase activity, coupled to transmembrane movement of substances (3/0.04), endopeptidase inhibitor activity (2/0.01), and S-adenosylmethionine-dependent methyltransferase activity (2/0.02) (**Figure 6B** left). KEGG enrichment indicated that 473 genes were mainly enriched in the ABC transporters (3/0.00), mitogen-activated protein kinase (MAPK) signaling pathway–plant (2/0.00), and starch and sucrose metabolism (2/0.01) (**Figure 6B** right).

For trait AL, genes in the module MEdarkolivegreen (0.53/0.02) were selected for enrichment analysis. GO enrichment indicated that 129 genes in this module were mainly involved in cytochrome-c oxidase activity (3/0.00), response to auxin (2/0.01), and response to stimulus (1/0.01) (**Figure 6C** left). KEGG enrichment indicated that 129 genes were mainly enriched in tropane, piperidine, and pyridine alkaloid biosynthesis (2/0.00), alanine, aspartate, and glutamate metabolism (1/0.03), and aminoacyl-tRNA biosynthesis (1/0.05) (**Figure 6C** right).

### Expression patterns of candidate differentially expressed genes and hub genes

**Figure 7:**
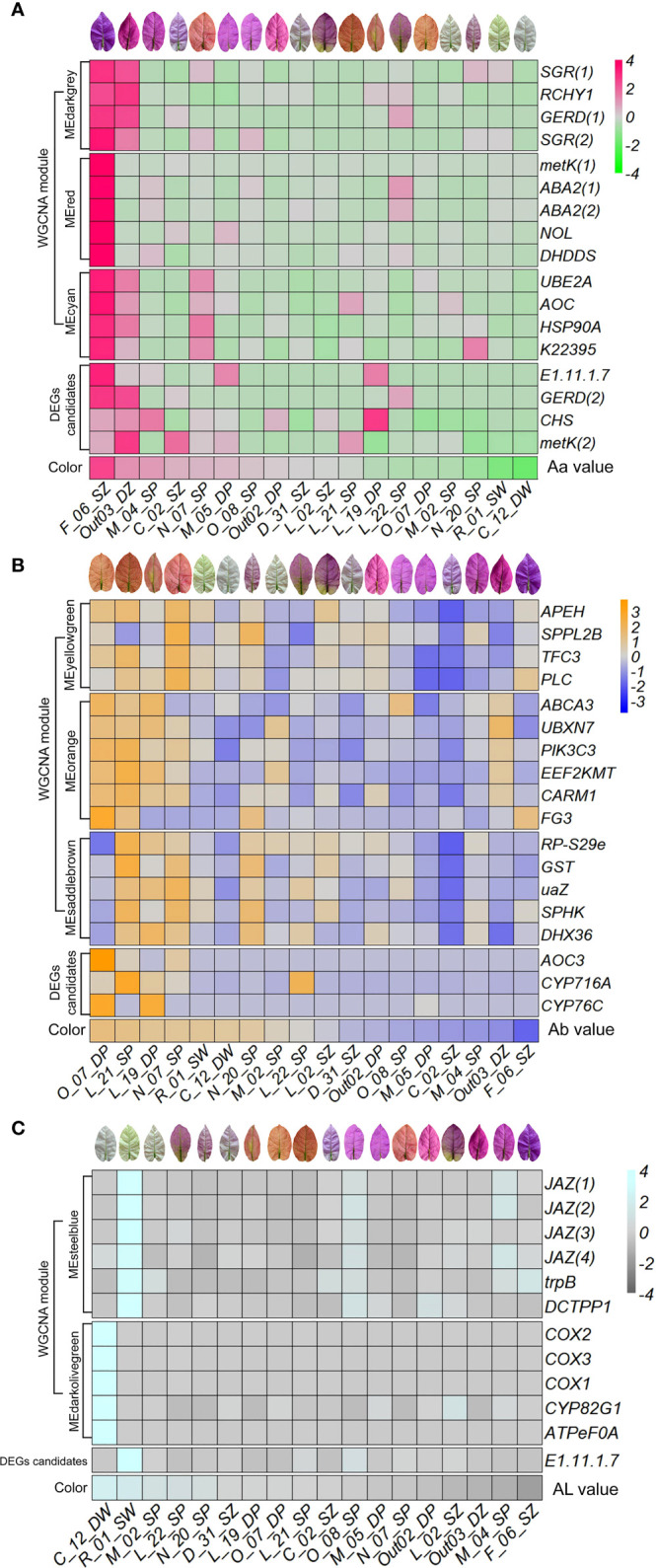
Expression patterns of selected DEG candidates and hub genes in each top WGCNA module along with bract color L-a-b values. Expression heatmap of selected DEG candidates and hub genes in each top WGCNA module along with color Aa **(A)**, Ab **(B)**, and AL **(C)** values ordered from high to low. The x axis represents each accession ordered by the color L-a-b values from high to low. The y axis represents DEG candidates, hub genes identified in each top module, as well as color trait values.

Candidate DEGs between accessions with high vs. low L-a-b values might potentially be involved in the regulation of bract coloration in *B. glabra*. In DEGs of high vs. low Aa values, one peroxidase 42 (*E1.11.1.7*) gene, one (-)-germacrene D synthase-like (*GERD*) gene, one chalcone synthase (*CHS*) gene, and one S-adenosylmethionine synthase 2-like (*metK*) gene were selected to show the consistency of expression variation with variation in bract Aa value (**Figure 7A**; **Supplementary Table S6**). In the accessions F_06_SZ, M_04_SP, and Out03_DZ with the highest Aa value, these genes were generally upregulated, while in the accessions N_20_SP, R_01_SW, and C_12_DW with lower Aa value, these genes were generally expressed less (**Figure 7A**). Moreover, in DEGs of high vs. low Ab values, one primary-amine oxidase (*AOC3*) gene, one cytochrome P450 (*CYP716A*) gene, and one cytochrome P450 76T24-like (*CYP76C*) gene were selected. In accessions O_07_DP, L_21_SP, L_19_DP, and N_07_SP with the highest Ab value, these genes were upregulated considerably, while in accessions F_06_SZ, Out03_DZ, M_04_SP, and C_02_SZ with lower Ab value, genes were generally downregulated (**Figure 7B**). For DEGs of high vs. low AL values, only *E1.11.1.7* gene was selected. In the accessions R_01_SW and C_12_DW with the highest AL value, *E1.11.1.7* was upregulated, while expressed less in other accessions (**Figure 7C**).

**Figure 8:**
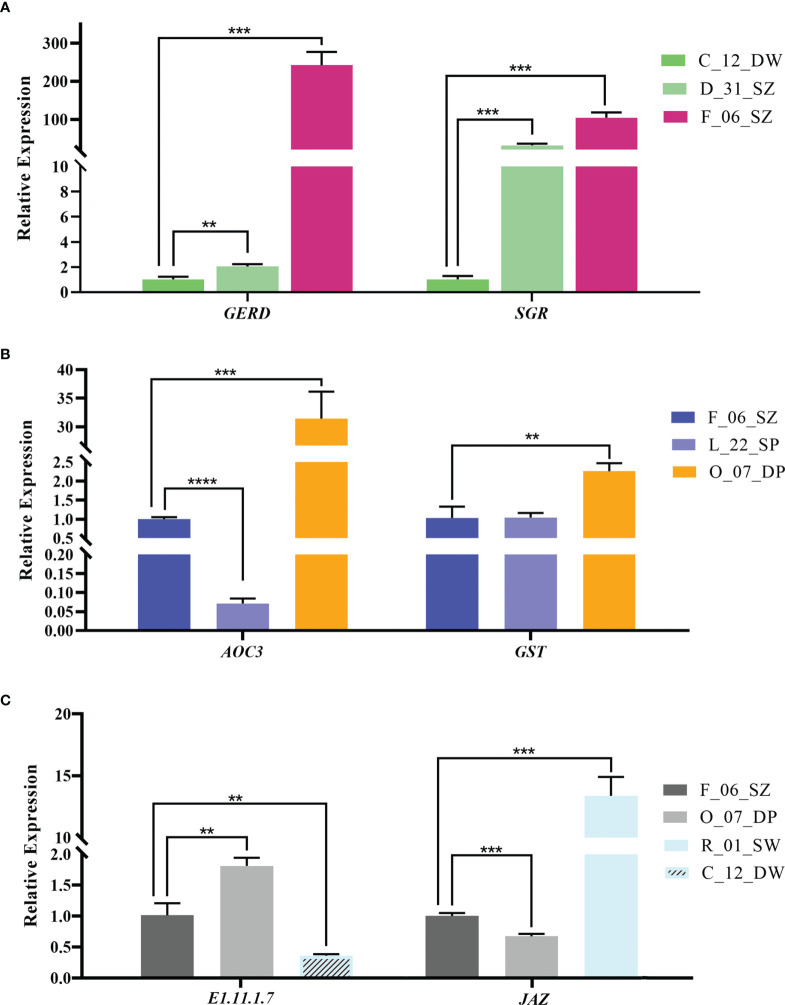
The relative expression of candidate genes in bracts of *Bougainvillea glabra* validated by RT-qPCR. **(A)** The expression patterns of genes *GERD* (from DEG analysis) and *SGR* (hub gene from the co-expression network) in accessions C_12_DW, D_31_SZ, and F_06_SZ following increased Aa value. **(B)** The expression patterns of genes *AOC3* (from DEG analysis) and *GST* (hub gene from the co-expression network) in accession F_06_SZ, L_22_SP, and O_07_DP following increased Ab value. **(C)** The expression patterns of genes *E1.11.1.7* (from DEG analysis) and *JAZ* (hub gene from the co-expression network) in accession F_06_SZ, O_07_DP, R_01_SW, and C_12_DW following increased AL value. Asterisks represent the significance of the *T*-test for each comparison as indicated by **P < 0.01, ***P < 0.001, and ****P < 0.0001.

In the top WGCNA module MEdarkgrey correlated with trait Aa, two stay-green protein (*SGR*) genes, RING finger and CHY zinc finger domain-containing protein 1 (*RCHY1*) gene, and one *GERD* were selected (**Figure 8A**; **Supplementary Table S6**). In module MEred, two xanthoxin dehydrogenase (*ABA2*) genes, one *metK*, ditrans,polycis-polyprenyl diphosphate synthase (*DHDDS*) gene, and chlorophyll b reductase (*NOL*) gene were selected to show the consistency of expression variation with variation in bract Aa value. In module MEcyan, one ubiquitin-conjugating enzyme E2 A (*UBE2A*) gene, one allene oxide cyclase (*AOC*) gene, one molecular chaperone HtpG (*HSP90A*) gene, and cinnamyl-alcohol dehydrogenase (*K22395*) gene were selected. In the accessions F_06_SZ and Out03_DZ with the highest Aa value, these genes were generally upregulated, while in the accessions R_01_SW and C_12_DW with lower Aa value, these genes were generally expressed less (**Figure 7A**).

For trait Ab in WGCNA module MEyellowgreen, one signal peptide peptidase-like 2B (*SPPL2B*) gene, one transcription factor C subunit 3 (*TFC3*) gene, one phospholipase C (*PLC*) gene, and one acylaminoacyl-peptidase (*APEH*) gene were selected to show the consistency of expression variation with variation in bract Ab value (**Figure 8B**; **Supplementary Table S6**). In module MEorange, ATP-binding cassette, subfamily A (*ABC1*) and member 3 (*ABCA3*), vacuolar protein sorting 34 (*PIK3C3*), one protein-lysine N-methyltransferase EEF2KMT (*EEF2KMT*) gene, one UBX domain-containing protein 7 (*UBXN7*) gene, one type I protein arginine methyltransferase (*CARM1*) gene, and one UDP-glycosyltransferase (*FG3*) gene were selected. In module MEsaddlebrown, one small subunit ribosomal protein S29e (*RP-S29e*) gene, one glutathione S-transferase T1 (*GST*) gene, one urate oxidase (*uaZ*) gene, one sphingosine kinase (*SPHK*) gene, and one ATP-dependent RNA helicase DHX36 (*DHX36*) gene were selected. In accessions O_07_DP, L_21_SP, L_19_DP, and N_07_SP with the highest Ab value, these genes were upregulated considerably, while in the accessions F_06_SZ, Out03_DZ, M_04_SP, and C_02_SZ with lower Ab value, genes were generally downregulated (**Figure 7B**).

For trait AL in module MEdarkolivegreen, three *COX1*, *COX2*, and *COX3*, one cytochrome P450 82G1 monooxygenase (*CYP82G1*) gene, and one F-type H+-transporting ATPase subunit a (*ATPeF0A*) gene were selected to show the consistency of expression variation with variation in bract AL value (**Figure 7C**; **Supplementary Table S6**). In module MEsteelblue, four jasmonate ZIM domain-containing protein (*JAZ*) genes, one dCTP diphosphatase (*DCTPP1*) gene, and one tryptophan synthase beta chain (*trpB*) gene were selected. In the accessions R_01_SW and C_12_DW with the highest AL value, these genes were generally upregulated, while genes were generally expressed in low levels in other accessions (**Figure 7C**).

### RT-qPCR validation

Among genes screened from DEG analysis, a *GERD* gene (TRINITY_DN19569_c0_g1) upregulated in accessions of high L-a-b values was chosen for RT-qPCR validation in C_12_DW, D_31_SZ, and F_06_SZ accessions following increased Aa value (**Figure 8A** left). RT-qPCR result showed that the trend of *GERD* gene expression was similar as that in transcriptome expression analysis (**Figures 7A**, **8A** left). Compared with C_12_DW and D_31_SZ, the F_06_SZ (*T*-test, *P* < 0.01; *P* < 0.001) exhibited increased expression of *GERD*. An *AOC3* gene (TRINITY_DN6496_c0_g2) upregulated in high Ab value accessions was also selected for RT-qPCR validation in F_06_SZ, L_22_SP, and O_07_DP accessions following increased Ab value (**Figure 8B** left). Results showed a reduced expression in L_22_SP (*T*-test, *P* < 0.0001) compared with F_06_SZ and an increased expression in O_07_DP (*T*-test, *P* < 0.001) compared with F_06_SZ. A peroxidase (*E1.11.1.7*) gene (TRINITY_DN1078_c0_g1) upregulated in high L value accessions was chosen to perform RT-qPCR validation in F_06_SZ, O_07_DP, and C_12_DW accessions following increased AL value (**Figure 8C** left). The results showed an increased expression in O_07_DP (*T*-test, *P* < 0.01) compared with F_06_SZ and a reduced expression in C_12_DW (*T*-test, *P* < 0.01) compared with F_06_SZ.

For the hub genes from each co-expression network, *SGR* gene (TRINITY_DN12271_c0_g2) was chosen for RT-qPCR validation in C_12_DW, D_31_SZ, and F_06_SZ accessions following increased Aa value (**Figure 8A** right). The trend of relative expression was consistent with the trend in transcriptome expression analysis (**Figures 7A**, **8A** right). Compared with C_12_DW, *SGR* was significantly upregulated (*T*-test, *P* < 0.001) in F_06_SZ and D_31_SZ accessions. *GST* gene (TRINITY_DN671_c0_g2) was selected for RT-qPCR validation in F_06_SZ, L_22_SP, and O_07_DP accessions following increasing Ab value. The expression of *GST* in O_07_DP (*T*-test, *P* < 0.01) was increased compared with F_06_SZ but no significant difference between F_06_SZ and L_22_SP (**Figure 8B** right). *JAZ* gene (TRINITY_DN16399_c0_g1) was also selected for RT-qPCR validation in F_06_SZ, O_07_DP, and R_01_SW accessions following increased AL value. The *JAZ* gene showed reduced expression in O_07_DP (*T*-test, *P* < 0.001) compared with F_06_SZ but significant upregulation in R_01_SW compared with F_06_SZ (*T*-test, *P* < 0.001) (**Figure 8C** right).

## Discussion

As a non-flower metamorphic organ in angiosperms, bracts are often neglected. However, *Bougainvillea* shows extensive color variation in bract color and shape across various cultivars, making it an ideal system for studying the neo-functionalization of bract coloration. Lack of genome information for *Bougainvillea* warranted the application of a pan-transcriptome-based approach. In this study, we have employed a pan-transcriptome assembly based on the transcriptome data of the 18 *B. glabra* accessions and revealed their germplasm kinship and regulation network of bract color variation. The variation of the bract color on the scale of L-a-b value was determined. In addition, by constructing the germplasm kinship of *B. glabra* accession using PCA, phylogenetic tree, and admixture analysis, three optimal subgroups were clustered, which were not directly correlated with bract color variation at the population level. Moreover, DEG analysis between accessions in high vs. low L-a-b value excavated hundreds of upregulated genes that might be involved in bract color variation of *B. glabra*. Finally, eight co-expressed regulation modules were identified by WGCNA, which have a high correlation with bract color variation of L-a-b values. Several hub genes in the co-expressed network were determined as the core regulation factors that might contribute to the variation of *B. glabra* bract coloration.

Analysis of bract color variation in CIE L-a-b values among the 18 accessions showed that the purple coloration of bracts was constituted with Aa+ and Ab- value, whereas the orange coloration was constituted with Aa+ and Ab+ value. Several studies showed that the pigmentation of *Bougainvillea* bracts are involved in betalain metabolism pathway, while other plant colorations were being linked to betalain biosynthesis (Gandiía-Herrero et al., 2005; Khan and Giridhar, 2015; Xu et al., 2016; Wu et al., 2022). Wu et al. (2022) recently showed that bract color variation of *Bougainvillea* is the result of varied proportions of betacyanins and betaxanthins. And their measurements of bract L-a-b value are consistent with our results. However, these studies only focused on the metabolism or pigmentation and did not investigate their coloration variation at the genome-wide population level. Population genomic study of *B. glabra* germplasm kinship showed three optimal subgroups among the 18 accessions with one subgroup relatively containing pure stratification and others were mixture structure. Although the kinship clustering of these accessions was not directly related to their bract coloration, we speculated that the color variation of *B. glabra* accessions might be due to the adaptation to pollinator selection in local environments. However, we do not exclude the exception of hybridization or genomic introgression during evolution or cultivation (Kobayashi et al., 2007; Ohri, 2013; Priyadarshini et al., 2017).

Either transcriptomes or metabolomes have been used to study the variation of specialized metabolites in *Bougainvillea* (Xu and Lin, 2010; Xu et al., 2016; Kumar et al., 2017; Lin et al., 2021; Ohno et al., 2021; Wu et al., 2022; Zhang et al., 2022). However, a comprehensive association study of gene expression and phenotypes has not been performed at the population scale. By integrating the co-expression profiles with bract phenotypical coloration data, we detected the positive correlation of the gene expression with bract color variation in *B. glabra* population. The 1,621 genes in WGCNA modules correlated with color Aa value enriched in a significant pathway of the response to UV-B. The pathway contained three UV-B receptor (*UVR8*) genes that were involved in the flavone, flavonol, and betalain biosynthesis (Tilbrook et al., 2013; Li et al., 2020; Ortega-Hernández et al., 2020) and might have an association with the variation of *B. glabra* bract color Aa values. Moreover, two abscisic-aldehyde oxidase (*AAO3*) genes found in these modules that participated in the carotenoid biosynthesis might also be involved in these processes (Colasuonno et al., 2017; Xia et al., 2020). Additionally, the 473 genes in WGCNA modules that correlated with color Ab value included three genes enriched in pathways of ABC transporters and a few *GST* genes that encoded glutathione-S-transferase, which might contribute to color variation and intensity of bract Ab coloration. It was reported that ABC transporter A family member (*ABCA*) genes participate in flavonoid transport (Sugiyama et al., 2007; Zhao and Dixon, 2010), while the regulation of GST-ABC transporters on flower color intensity was reported in carnations (Sasaki et al., 2012; Vilperte et al., 2021). Furthermore, 129 WGCNA module genes highly correlated with color AL value and were mostly enriched in cytochrome-c oxidase activity. Among them, three notable cytochrome c oxidase subunit I, II, III (*COX1*, *COX2*, *COX3*) genes involved in oxidative phosphorylation during fruit ripening and petal senescence (Considine et al., 2001; Azad et al., 2008; Song et al., 2015) might be related to bract color AL traits controlling the bract brightness changes.

The bract color of *B. glabra* is one of the most important phenotypic traits affecting its ornamental value. The multicolored bracts of *B. glabra* are mainly attributed to pigments: betalains, carotenoids, flavonoids, and chlorophylls (Xu and Lin, 2010; Ahmed, 2014; Kumar et al., 2017; Mahendran et al., 2020). According to the expression patterns of DEG candidates and co-expressed hub genes in the green to red dimension (Aa value) of bract color, two *GERD* genes and two *SGR* genes were identified. *GERD* gene encodes germacrene D, considered as a precursor of many sesquiterpenes (Bülow and König, 2000). A previous study showed that pollinator-mediated selection on floral traits can occur simultaneously, leading to specific scent–color combinations as the pollination syndromes (Fenster et al., 2004). Several studies also revealed the pigment–scent connection in anthocyanin synthesis plants (Majetic et al., 2010; Yeon and Kim, 2020; Yeon and Kim, 2021), but few have focused on betalain synthesis in plants (Majetic et al., 2010). We found that the trend of *GERD* gene expression was significantly upregulated following increased Aa value, which was also validated in RT-qPCR experiment. *SGR* de-chelates magnesium from chlorophyll a and induces the chlorophyll b reductase *NOL* expression to activate the degradation of chlorophyll b, thereby achieving the degradation of chlorophyll, resulting in green color reduction in leaves (Sato et al., 2018). Shen et al. (2018) found that upregulated *SGR* might have changed the color of tea leaf from green to purple. Both expression pattern analysis and RT-qPCR experiment indicated that two *SGR* genes and a *NOL* showed high expression in accessions with the highest Aa value. Notably, we observed upregulation of major carotenoid biosynthesis pathway gene *ABA2*, which was consistent with increased Aa value, especially in accession F_06_SZ. It might be possible that the downregulation of *SGR*, *NOL*, and *ABA2* genes led to the higher concentrations of chlorophyll and lower concentrations of carotenoids, resulting in green bract coloration (Gonzaílez-Guzmaín et al., 2002). Barrero (2008) found that the ABA type gene mutation showed impaired production of the β-carotene-derived xanthophylls, neoxanthin, violaxanthin, and antheraxanthin and resulted in skotomorphogenic phenotype. When these three genes were at high expression levels, chlorophyll might be decreased and carotenoids might be accumulated, leading to the formation of red and purple coloration in *Bougainvillea* bracts (Sato et al., 2018). Moreover, one *E1.11.1.7* involved in decolorization of betacyanins and betaxanthin in color modifications in betalain biosynthesis plant was also observed (Josefa et al., 1998; Kugler et al., 2007; Wasserman et al., 2010; Manchali et al., 2012; Pedre O and Escribano, 2000). The Aa value variation might be due to the better antiradical activity of betacyanins than that of the betaxanthins in *B. glabra* bracts.

Although *B. glabra* is dominated by betalains, there are also flavonoids such as quercetin, kaempferol, myricetin, apigenin, and isorhamnetin (Xu and Lin, 2010; Ahmed, 2014; Maran et al., 2015). In the yellow to blue dimension (Ab value) of bract color, we identified a hue gene *FG3* involved in flavone and flavonol biosynthesis with UDP-glycosyltransferase activity (Di et al., 2015). *FG3* was upregulated at orange bract accession O_07_DP and L_21_SP, which had the highest Ab value, while *FG3* was also highly expressed in F_06_SZ, the individual with the lowest Ab value (**Figure 7B**). The result indicated that gene regulations of flavonoid metabolism might contribute to the intensity of coloration, making the bracts appear orange and purple-blue combined with other pigments. A study showed that *FG3* might cause a reduction of photosynthesis when normal flavonol levels are being produced (Buttery and Buzzell, 1987). Specifically, both *GST* and *ABCA3* genes showed high expression mainly in highest Ab value individuals, which was validated by RT-qPCR. The *GST-ABC* transporters and endoplasmic reticulum mediation have recently been reported to be linked to secondary metabolite transportation, such as trafficking anthocyanins in *Arabidopsis*, carnation, and maize (Sugiyama et al., 2007; Sasaki et al., 2012; Vilperte et al., 2021). These two genes might also be involved in betalain transport and be responsible for the intensity of bract color in *Bougainvillea*. Additionally, several studies showed that *CYP76AD5/6/1* is required for the initial tyrosine hydroxylation to *L-DOPA*, and *CYP76AD1* is required for the conversion of *L-DOPA* to *cDOPA* in beet betalain biosynthesis (Tanaka et al., 2008; Gandía-Herrero and García-Carmona, 2013; Ohno et al., 2021; Tossi et al., 2021). Our findings of high Ab value-biased expressed DEG genes *CYP716A* and *CYP76C* encoding cytochrome P450 might participate in betalain coloration of *B. glabra* bract.

It is interesting that several co-expressed genes correlated with the bract brightness dimension (AL value) were enriched in MAPK signaling pathway, with four *JAZ* genes as the hub genes. The expression of four *JAZ* genes was significantly upregulated in the brightest white accession R_01_SW and downregulated in the darkest purple accession F_06_SZ, which was also validated by RT-qPCR. The surface brightness of bracts is mainly related to their texture and trichome structure (Pierce et al., 2001; Yang et al., 2015; Raphael et al., 2017). Studies demonstrate that in *Arabidopsis*, both DELLAs and JAZs interacted with the WD-repeat/bHLH/MYB complex to mediate the synergistic and mutually dependent action between GA and jasmonate signaling in regulating plant trichome development (Qi et al., 2014; Lloyd et al., 2017). In addition, we found that three *COX* genes encoding cytochrome c oxidase subunits I, II, and III along with a cytochrome P450 82G1 (*CYP82G1*) as the hub genes showed high expression in brightest white accession C_12_DW. Several studies showed that *COX* protein accumulated in ripping fruits and petal senescence (Considine et al., 2001; Azad et al., 2008; Song et al., 2015). The *CYP82G1* involved in volatile homoterpene synthase (Sungbeom et al., 2010) might act as the factor of scent–color combinations, expressed highly in accessions of white than purple bract. The roles of *COX* hub genes and *CYP82G1* gene in the regulation of *B. glabra* bract surface brightness need future validation. Moreover, *E1.11.1.7* involved in decolorization of betacyanins and betaxanthin showed high expression in white bract accessions that might be due to the betalains (e.g., betacyanins) and can be decolorized by peroxidase (Josefa et al., 1998; Kugler et al., 2007; Wasserman et al., 2010).

## Conclusion

In this study, a pan-transcriptome was employed to study the germplasm kinship and regulation network of bract color variation in *B. glabra* at the population level. The bract color variation among the different accessions of *B. glabra* was quantified in continuous CIE L-a-b values. The germplasm kinship showed that *B. glabra* accessions clustered into three subgroups with two of them distinctly clustered but not directly related to color variation. The pan-transcriptome-level DEG analysis and co-expression network of the 18 accessions of *B. glabra* were achieved. Several DEG candidates and hub genes (e.g., *GERD*, *SGR*, *ABCA3*, *GST*, *CYP76AD1*, *CYP76C*, and *JAZ*) in the co-expression network were determined that might be involved in regulating the *B. glabra* bract color variation of L-a-b values. Our research will provide the important foundation for the studies into the evolution and regulation mechanism of bract traits at the population level with the pan-transcriptome, as well as the application of ornamental traits in horticultural plants.

## Data availability statement

The datasets used in this study can be found in online repositories. The names of the repository/repositories and accession number(s) can be found in the article/**Supplementary Material**.

## Author contributions

XM and JY conceived the project and designed the experiments, HH, HJ, SJ, WL, and LG collected the samples. HH, HJ, WL, and JL conducted the experiments, HH, HJ, SJ, and XM processed and analyzed the data. SJ, XL, and LL visualized the experimental results. JL, HH, and XL conducted the RT-qPCR experiments. HH and XM wrote the manuscript. XM, HJ, and DQ revised the manuscript. All authors read and approved the final manuscript.

## Funding

This work has been supported by grant from The Earmarked Fund of Science and Technology Innovation for Fujian Agriculture and Forestry University to XM (Project No. KFb22112XA), The National Natural Science Foundation of China to XM (Project No. 31700199), and The Earmarked Fund for Jiangxi Agriculture Research System to HJ (Project No. JXARS-17).

## Conflict of interest

The authors declare that the research was conducted in the absence of any commercial or financial relationships that could be construed as a potential conflict of interest.

## Publisher’s note

All claims expressed in this article are solely those of the authors and do not necessarily represent those of their affiliated organizations, or those of the publisher, the editors and the reviewers. Any product that may be evaluated in this article, or claim that may be made by its manufacturer, is not guaranteed or endorsed by the publisher.
