# Supplementary material for: Pantranscriptome combined with phenotypic quantification reveals germplasm kinship and regulation network of bract color variation in Bougainvillea
Source: Front Plant Sci. 2022 Nov 17;13:1018846. doi: 10.3389/fpls.2022.1018846 (PMC9713818; doi:10.3389/fpls.2022.1018846)
Supplement: Supplementary file 1 [file DataSheet_1.pdf]

Supplementary materials

Data Source

All RNA-seq data and transcriptome assembly of *Bougainvillea glabra* used in this paper was downloaded from the Genome Sequence Archive (GSA), National Genomics Data Center (NGDC) under BioProject number PRJCA011746.

Supplementary Table 1. Information about 18 *B. glabra* accessions.

Accessions	Species	Cultivar	Bract color	Bract shape
C_02_SZ	*B. glabra*	Formosa	light purple	oval
C_12_DW	*B. glabra*	White Stripe	white	long ellipse
D_31_SZ	*B. glabra*	Brasiliensis	light purple	oval
F_06_SZ	*B. glabra*	John Lattin	deep purple	ellipse to oval
L_02_SZ	*B. glabra*	Whisper	purple	oval
L_19_DP	*B. glabra*	Patch Pink	orange	long ellipse
L_21_SP	*B. glabra*	Bambino Pedero	orange	ellipse to oval
L_22_SP	*B. glabra*	Singapore Beauty	light purple	ellipse
M_02_SP	*B. glabra*	Autumn	light purple	ellipse to oval
M_04_SP	*B. glabra*	Dwarf Lavender	purple	oval
M_05_DP	*B. glabra*	Jarum	purple	ellipse to oval
N_07_SP	*B. glabra*	Australian Pink	red to orange	ellipse to oval
N_20_SP	*B. glabra*	Mini Cherry	light purple	long ellipse
O_07_DP	*B. glabra*	Kikori	orange	oval
O_08_SP	*B. glabra*	Dr. David Barry	purple	oval
Out02_DP	*B. glabra*	Mrs. E. W. Bick	purple	ellipse to oval
Out03_DZ	*B. glabra*	Glabra Variegated	deep purple	ellipse to oval
R_01_SW	*B. glabra*	Snow Queen	white	ellipse to oval

Supplementary Table 2. primers sequence of candidate genes used in RT-qPCR experiments.

Gene	Forward primers	Reverse primers
SGR	ATCAACCATAGAAAACTCCAGAACC	AAGAAGAAATCAATCAGCGGC
GST	TTCTCTCCTGATTTACCGTTCTTC	TCGGCTTTTGTGCCTCCTAT
AOC3	TCCTCTTTTCTCTTCTTTCACCG	AGCAGCACACAAATTGGCTC
JAZ	GGGCTAAAGAGATATGTGGAGCA	ATCAGAACTGACGGCAGATGAT
GERD	CTACTACTCTTCGGTTTCGGCTA	GGCTTCTTCTAATATGTCTTCTCCA
E1.11.1.7	CCATCCTTCTCGGCTTTGTT	GACTGCTTCATTAGGGTATGTTTTC
18S rRNA	CAGAACATCTAAGGGCATCACA	TAGTTGGTGGAGCGATTTGTCT

Supplementary Table 3. L-a-b color values of bracts from 18 *B. glabra* accessions.

Accessions	a (a*; mean±SD, n=4)	b (b*; mean±SD, n=4)	L (L*; mean±SD, n=4)
C_02_SZ	24.38±1.46	-13.06±0.93	54.21±0.36
C_12_DW	-1.98±0.35	8.82±0.96	67.9±1.04
D_31_SZ	17.84±0.56	-7.95±0.50	55.82±0.86
F_06_SZ	44.81±3.82	-24.88±2.25	43.04±2.32
L_02_SZ	17.24±3.43	-4.61±3.77	48.38±2.04
L_19_DP	12.15±1.45	10.52±0.82	55.2±2.04
L_21_SP	15.82±1.28	13.23±1.55	54.6±0.71
L_22_SP	11.25±3.70	-0.21±3.02	59.1±2.08
M_02_SP	10.68±0.25	2.41±0.18	60.84±0.85
M_04_SP	27.89±1.95	-14.78±1.59	46.35±2.19
M_05_DP	22.14±0.73	-11.59±0.74	51.72±1.03
N_07_SP	22.75±3.11	9.84±1.36	51.41±2.54
N_20_SP	7.6±0.79	8.03±0.10	58.5±0.73
O_07_DP	11.08±0.48	15.38±1.69	55.06±1.13
O_08_SP	20.5±2.30	-10.77±1.63	53.31±1.07
Out02_DP	18.72±1.32	-8.97±0.63	50.72±3.64
Out03_DZ	29.75±1.86	-16.2±2.02	47.78±0.71
R_01_SW	1.12±1.08	9.5±2.18	64.66±1.69

Supplementary Table 4. Up regulated genes in DEG analysis for contrast comparisons of bracts L-a-b values.

Lab	Gene_id	NR description	Log2FC	Padjust
A	TRINITY_DN6190_c0_g1	-	8.33	0.00
A	TRINITY_DN6601_c0_g1	mannose/glucose-specific lectin-like	7.15	0.00
A	TRINITY_DN8975_c1_g1	peroxidase 42	5.90	0.00
A	TRINITY_DN17118_c0_g2	RecName: Full=Antimicrobial peptide 1;	8.17	0.00
A	TRINITY_DN19569_c0_g1	(-)-germacrene D synthase-like	5.94	0.00
A	TRINITY_DN20832_c0_g1	kunitz trypsin inhibitor 2-like	4.73	0.00
A	TRINITY_DN12022_c1_g4	PREDICTED: stomatal closure-related actin-binding protein 3	5.60	0.00
A	TRINITY_DN902_c0_g1	PREDICTED: (R,S)-reticuline 7-O-methyltransferase	5.20	0.00
A	TRINITY_DN19419_c0_g1	-	8.03	0.00
A	TRINITY_DN7418_c0_g1	cysteine-rich receptor-like protein kinase 10	5.39	0.00
A	TRINITY_DN40851_c0_g1	-	3.37	0.00
A	TRINITY_DN133461_c0_g2	-	7.98	0.00
A	TRINITY_DN56405_c0_g1	-	2.62	0.00
A	TRINITY_DN543_c0_g3	-	7.40	0.00
A	TRINITY_DN8697_c0_g2	-	4.49	0.00
A	TRINITY_DN12005_c0_g2	-	5.28	0.00
A	TRINITY_DN5423_c0_g2	-	7.94	0.00
A	TRINITY_DN8328_c1_g3	-	7.58	0.00
A	TRINITY_DN49373_c0_g1	(R,S)-reticuline 7-O-methyltransferase-like	5.99	0.01
A	TRINITY_DN7172_c0_g2	probable pectate lyase 8	3.07	0.01
A	TRINITY_DN156_c0_g2	-	4.16	0.01
A	TRINITY_DN6551_c0_g1	-	5.42	0.01
A	TRINITY_DN2581_c0_g1	-	2.34	0.01
A	TRINITY_DN62788_c0_g1	proteinase inhibitor I3	2.98	0.01
A	TRINITY_DN34428_c0_g1	S-adenosylmethionine synthase 2-like	4.81	0.01
A	TRINITY_DN12962_c0_g1	polygalacturonase inhibitor 1-like	3.37	0.01
A	TRINITY_DN2698_c0_g1	MLP-like protein 43	2.57	0.01
A	TRINITY_DN122426_c0_g1	-	5.31	0.01
A	TRINITY_DN134954_c0_g1	chalcone synthase	2.56	0.01
A	TRINITY_DN4447_c0_g1	-	6.23	0.01
A	TRINITY_DN15371_c0_g1	-	3.00	0.01
A	TRINITY_DN26790_c1_g1	-	4.81	0.01
A	TRINITY_DN9686_c0_g1	probable aspartyl protease At4g16563	2.61	0.01
A	TRINITY_DN90372_c0_g1	PREDICTED: disease resistance protein At4g27190 isoform X2	7.63	0.01
A	TRINITY_DN129672_c0_g1	-	7.58	0.01
A	TRINITY_DN43846_c0_g1	cysteine-rich receptor-like protein kinase 15	4.63	0.01
A	TRINITY_DN20997_c0_g1	-	6.62	0.01
A	TRINITY_DN16844_c0_g1	probable pectinesterase 53	4.87	0.01
A	TRINITY_DN201_c4_g1	-	7.23	0.02
A	TRINITY_DN73120_c0_g1	PREDICTED: L10-interacting MYB domain-containing protein	5.14	0.02
A	TRINITY_DN9494_c0_g1	-	7.52	0.02
A	TRINITY_DN10624_c0_g1	-	3.32	0.02
A	TRINITY_DN7582_c0_g1	-	2.75	0.02
A	TRINITY_DN26685_c0_g1	-	6.68	0.02
A	TRINITY_DN39195_c0_g1	secreted RxLR effector protein 161-like	4.51	0.02
A	TRINITY_DN35283_c0_g2	-	6.13	0.02
A	TRINITY_DN23375_c0_g1	-	4.98	0.03
A	TRINITY_DN30783_c1_g1	-	7.01	0.03
A	TRINITY_DN34950_c0_g1	-	6.99	0.03
A	TRINITY_DN12805_c0_g1	-	4.02	0.03
A	TRINITY_DN105984_c0_g1	-	3.54	0.03
A	TRINITY_DN35926_c0_g1	LOW QUALITY PROTEIN: glutathione hydrolase 1-like	4.79	0.03
A	TRINITY_DN5459_c0_g1	-	3.92	0.03
A	TRINITY_DN75591_c0_g1	-	6.86	0.03

A	TRINITY_DN3381_c0_g1	flavin-containing monooxygenase FMO GS-OX5-like	3.14	0.03
A	TRINITY_DN21184_c0_g1	putative glycine-rich cell wall structural protein 1	5.27	0.03
A	TRINITY_DN15817_c0_g1	-	4.36	0.03
A	TRINITY_DN8472_c0_g1	probable xyloglucan endotransglucosylase/hydrolase protein 23	3.33	0.03
A	TRINITY_DN672_c1_g1	-	2.61	0.03
A	TRINITY_DN37650_c0_g1	-	4.57	0.03
A	TRINITY_DN25348_c0_g1	cysteine protease RD19A-like	7.02	0.03
A	TRINITY_DN1469_c2_g1	-	3.10	0.04
A	TRINITY_DN87178_c0_g1	-	3.67	0.04
A	TRINITY_DN19218_c0_g2	-	6.94	0.04
A	TRINITY_DN39393_c0_g1	-	3.63	0.04
A	TRINITY_DN47623_c0_g1	-	7.51	0.04
A	TRINITY_DN109371_c0_g1	uclacyanin 1-like	3.16	0.04
A	TRINITY_DN6180_c0_g1	PREDICTED: GDSL esterase/lipase At5g14450	2.18	0.04
A	TRINITY_DN3658_c0_g2	germin-like protein subfamily 1 member 15	3.50	0.04
A	TRINITY_DN136774_c0_g1	3-epi-6-deoxocathasterone 23-monooxygenase	3.51	0.04
A	TRINITY_DN108677_c0_g1	-	11.23	0.04
A	TRINITY_DN64474_c0_g1	L-ascorbate oxidase homolog	2.38	0.04
A	TRINITY_DN40432_c0_g2	-	5.84	0.05
A	TRINITY_DN14618_c0_g1	-	3.48	0.05
A	TRINITY_DN18245_c0_g1	phosphorylase domain-containing protein, partial	4.69	0.05
A	TRINITY_DN29879_c0_g1	protein FAR1-RELATED SEQUENCE 1-like	7.07	0.05
B	TRINITY_DN3787_c0_g1	protein NLP6-like, partial	5.38	0.00
B	TRINITY_DN2414_c0_g5	-	10.00	0.00
B	TRINITY_DN12941_c0_g1	-	6.27	0.00
B	TRINITY_DN12696_c0_g1	-	9.51	0.00
B	TRINITY_DN4187_c0_g2	-	9.40	0.00
B	TRINITY_DN1311_c0_g1	-	6.67	0.00
B	TRINITY_DN8281_c1_g1	-	9.11	0.00
B	TRINITY_DN19858_c2_g4	-	7.03	0.00
B	TRINITY_DN2643_c1_g1	-	9.96	0.00
B	TRINITY_DN4285_c0_g1	PREDICTED: albumin-2	10.66	0.00
B	TRINITY_DN7528_c0_g2	-	8.13	0.00
B	TRINITY_DN15653_c0_g4	Integrase catalytic core	8.23	0.00
B	TRINITY_DN4885_c0_g2	-	8.81	0.00
B	TRINITY_DN6496_c0_g2	-	10.13	0.00
B	TRINITY_DN4548_c0_g1	-	6.26	0.00
B	TRINITY_DN63034_c0_g1	root phototropism protein 2-like	5.94	0.00
B	TRINITY_DN127986_c1_g2	PREDICTED: cyclic nucleotide-gated ion channel 17 isoform X1	8.09	0.00
B	TRINITY_DN75196_c0_g1	PREDICTED: UBP1-associated protein 2A	8.63	0.00
B	TRINITY_DN80318_c0_g1	-	7.69	0.00
B	TRINITY_DN58234_c1_g1	-	7.69	0.00
B	TRINITY_DN90447_c0_g1	-	7.97	0.00
B	TRINITY_DN92652_c0_g1	-	8.14	0.00
B	TRINITY_DN77314_c0_g4	-	7.53	0.00
B	TRINITY_DN10475_c0_g1	replication protein A 70 kDa DNA-binding subunit B-like	7.82	0.00
B	TRINITY_DN34154_c0_g6	putative ribonuclease H protein	7.40	0.00
B	TRINITY_DN3465_c0_g3	-	8.41	0.00
B	TRINITY_DN11659_c0_g1	-	8.22	0.00
B	TRINITY_DN4885_c0_g3	-	8.26	0.00
B	TRINITY_DN5042_c1_g1	-	5.15	0.00
B	TRINITY_DN17073_c1_g1	-	3.73	0.00
B	TRINITY_DN15647_c0_g3	-	7.23	0.00
B	TRINITY_DN12035_c0_g3	-	6.72	0.00
B	TRINITY_DN130341_c0_g1	-	4.58	0.00
B	TRINITY_DN24158_c0_g1	cytochrome P450	4.73	0.01
B	TRINITY_DN52497_c0_g1	-	7.40	0.01
B	TRINITY_DN15365_c0_g1	-	7.20	0.01
B	TRINITY_DN99293_c0_g1	-	7.50	0.01
B	TRINITY_DN4281_c0_g2	-	7.83	0.01
B	TRINITY_DN10285_c1_g2	-	7.04	0.01

B	TRINITY_DN2323_c1_g3	-	6.19	0.01
B	TRINITY_DN17181_c0_g1	pectinesterase	7.09	0.01
B	TRINITY_DN127986_c1_g1	PREDICTED: cyclic nucleotide-gated ion channel 17 isoform X2	6.95	0.01
B	TRINITY_DN41301_c0_g1	cytochrome P450 76T24-like	6.86	0.02
B	TRINITY_DN773_c0_g2	-	7.00	0.02
B	TRINITY_DN67941_c0_g2	-	6.75	0.02
B	TRINITY_DN27222_c1_g1	disease resistance protein RGA2-like	4.07	0.02
B	TRINITY_DN31334_c0_g2	-	7.32	0.02
B	TRINITY_DN93806_c0_g2	-	6.73	0.02
B	TRINITY_DN5546_c2_g1	-	3.56	0.02
B	TRINITY_DN61629_c0_g2	-	6.96	0.02
B	TRINITY_DN12370_c1_g1	putative mitochondrial protein	6.78	0.02
B	TRINITY_DN66167_c0_g1	-	6.23	0.02
B	TRINITY_DN27147_c0_g1	disease resistance protein RGA2-like	4.72	0.02
B	TRINITY_DN125819_c1_g1	-	6.71	0.02
B	TRINITY_DN27900_c0_g2	-	4.94	0.02
B	TRINITY_DN4072_c0_g3	probable inactive purple acid phosphatase 29	3.35	0.02
B	TRINITY_DN33708_c1_g2	-	6.26	0.03
B	TRINITY_DN1399_c0_g2	-	6.56	0.03
B	TRINITY_DN18631_c0_g3	-	6.08	0.03
B	TRINITY_DN19895_c0_g2	PREDICTED: epoxide hydrolase 4	3.41	0.03
B	TRINITY_DN35638_c0_g2	-	5.24	0.04
B	TRINITY_DN1409_c2_g1	-	5.42	0.04
B	TRINITY_DN49512_c0_g1	-	5.73	0.04
B	TRINITY_DN30266_c0_g2	-	6.45	0.04
B	TRINITY_DN19005_c3_g1	-	7.21	0.04
B	TRINITY_DN10806_c1_g1	-	6.69	0.04
B	TRINITY_DN108621_c0_g1	-	6.62	0.04
B	TRINITY_DN11085_c0_g2	-	5.63	0.04
B	TRINITY_DN93806_c0_g3	-	6.46	0.04
B	TRINITY_DN67570_c0_g1	-	4.62	0.04
B	TRINITY_DN30274_c0_g1	-	4.09	0.05
B	TRINITY_DN40357_c0_g1	-	4.79	0.05
B	TRINITY_DN10539_c0_g2	-	5.73	0.05
B	TRINITY_DN56458_c1_g2	-	7.05	0.05
B	TRINITY_DN81120_c0_g1	-	5.22	0.05
L	TRINITY_DN3536_c0_g1	-	6.34	0.00
L	TRINITY_DN5705_c0_g1	PREDICTED: probable cyclin-dependent serine/threonine-protein kinase DDB_G0292550	6.71	0.00
L	TRINITY_DN51828_c0_g1	-	3.31	0.00
L	TRINITY_DN123406_c0_g1	-	5.69	0.00
L	TRINITY_DN10341_c0_g1	vacuolar-processing enzyme alpha-isozyme-like	3.29	0.00
L	TRINITY_DN25481_c0_g1	-	5.12	0.00
L	TRINITY_DN92719_c0_g1	-	7.48	0.00
L	TRINITY_DN107455_c0_g1	ent-copalyl diphosphate synthase, chloroplastic-like isoform X2	5.12	0.00
L	TRINITY_DN8108_c0_g1	ATP binding protein, putative	2.50	0.01
L	TRINITY_DN9312_c0_g1	-	4.25	0.01
L	TRINITY_DN59858_c1_g1	-	7.40	0.01
L	TRINITY_DN9531_c0_g1	-	7.40	0.01
L	TRINITY_DN4924_c0_g1	-	4.47	0.01
L	TRINITY_DN2827_c0_g1	diacylglycerol O-acyltransferase 3, cytosolic-like	2.02	0.01
L	TRINITY_DN50447_c0_g1	PREDICTED: laccase-15-like	7.62	0.01
L	TRINITY_DN14955_c0_g1	-	6.23	0.02
L	TRINITY_DN56458_c1_g2	-	6.78	0.02
L	TRINITY_DN14866_c0_g1	-	4.27	0.02
L	TRINITY_DN101594_c0_g1	transcription factor bHLH101-like	4.91	0.02
L	TRINITY_DN1078_c0_g1	peroxidase, partial	3.69	0.03
L	TRINITY_DN21455_c0_g1	thioredoxin H-type-like	2.77	0.03
L	TRINITY_DN4602_c0_g2	Retrovirus-related Pol polyprotein from transposon TNT 1-94	3.05	0.03
L	TRINITY_DN147268_c0_g1	-	4.34	0.03

Supplementary Table 5. Hub genes from top WGCNA modules correlated with bract color L-a-b values.

Lab	module	Gene ID	NR annotation
A	MEdarkgrey	TRINITY_DN12271_c0_g2	protein STAY-GREEN 1, chloroplastic-like
A	MEdarkgrey	TRINITY_DN21532_c0_g1	-
A	MEdarkgrey	TRINITY_DN10035_c0_g1	-
A	MEdarkgrey	TRINITY_DN13786_c0_g1	non-specific phospholipase C3-like
A	MEdarkgrey	TRINITY_DN39990_c0_g1	non-specific phospholipase C4-like
A	MEdarkgrey	TRINITY_DN17118_c0_g2	RecName: Full=Antimicrobial peptide 1; AltName: Full=MJ-AMP1; Short=AMP1; Flags: Precursor
A	MEdarkgrey	TRINITY_DN10051_c0_g1	-
A	MEdarkgrey	TRINITY_DN5459_c0_g1	-
A	MEdarkgrey	TRINITY_DN115809_c0_g1	delta-1-pyrroline-5-carboxylate dehydrogenase 12A1, mitochondrial-like
A	MEdarkgrey	TRINITY_DN19569_c0_g1	(-
A	MEdarkgrey	TRINITY_DN91018_c1_g1	-
A	MEdarkgrey	TRINITY_DN4102_c0_g1	-
A	MEdarkgrey	TRINITY_DN109371_c0_g1	uclacyanin 1-like
A	MEdarkgrey	TRINITY_DN5726_c0_g1	E3 ubiquitin-protein ligase MIEL1-like isoform X2
A	MEdarkgrey	TRINITY_DN51728_c0_g2	-
A	MEdarkgrey	TRINITY_DN1821_c1_g1	serine/threonine-protein phosphatase 7 long form homolog isoform X1
A	MEdarkgrey	TRINITY_DN39587_c0_g2	-
A	MEdarkgrey	TRINITY_DN9344_c0_g1	protein STAY-GREEN 1, chloroplastic-like
A	MEdarkgrey	TRINITY_DN14586_c0_g1	-
A	MEdarkgrey	TRINITY_DN33451_c0_g1	-
A	MEdarkgrey	TRINITY_DN71187_c1_g1	-
A	MEdarkgrey	TRINITY_DN10624_c0_g1	-
A	MEdarkgrey	TRINITY_DN11266_c0_g1	-
A	MEdarkgrey	TRINITY_DN13299_c0_g1	-
A	MEdarkgrey	TRINITY_DN15247_c0_g1	PREDICTED: DEAD-box ATP-dependent RNA helicase 35
A	MEdarkgrey	TRINITY_DN125831_c0_g1	Retrovirus-related Pol polyprotein from transposon TNT 1-94
A	MEdarkgrey	TRINITY_DN47575_c0_g1	boron transporter 1-like isoform X2
A	MEdarkgrey	TRINITY_DN15371_c0_g1	-
A	MEdarkgrey	TRINITY_DN7164_c1_g1	reverse transcriptase, partial
A	MEdarkgrey	TRINITY_DN2236_c0_g1	PREDICTED: UDP-glycosyltransferase 86A2-like
A	MEdarkgrey	TRINITY_DN15462_c0_g1	zinc finger BED domain-containing protein RICESLEEPER 2-like
A	MEcyan	TRINITY_DN24908_c0_g1	Vitis rotundifolia cultivar Noble chromosome 6
A	MEcyan	TRINITY_DN47558_c2_g1	TPA_asm: hypothetical protein HUJ06_005449
A	MEcyan	TRINITY_DN27816_c0_g1	-
A	MEcyan	TRINITY_DN112782_c0_g1	Retrovirus-related Pol polyprotein from transposon TNT 1-94
A	MEcyan	TRINITY_DN5217_c0_g1	-
A	MEcyan	TRINITY_DN39195_c0_g1	secreted RxLR effector protein 161-like
A	MEcyan	TRINITY_DN20874_c0_g1	Retrovirus-related Pol polyprotein from transposon TNT 1-94
A	MEcyan	TRINITY_DN5772_c0_g1	allene oxide cyclase, chloroplastic-like
A	MEcyan	TRINITY_DN19005_c1_g1	Transposon TX1 uncharacterized 149 kDa protein
A	MEcyan	TRINITY_DN47429_c0_g1	-
A	MEcyan	TRINITY_DN9765_c0_g1	-
A	MEcyan	TRINITY_DN119350_c0_g1	Retrovirus-related Pol polyprotein from transposon TNT 1-94

A	MEcyan	TRINITY_DN5422_c1_g1	-
A	MEcyan	TRINITY_DN74097_c0_g1	-
A	MEcyan	TRINITY_DN5273_c3_g1	-
A	MEcyan	TRINITY_DN57944_c0_g2	-
A	MEcyan	TRINITY_DN258_c5_g1	-
A	MEcyan	TRINITY_DN16579_c0_g1	F-box/kelch-repeat protein At3g06240-like
A	MEcyan	TRINITY_DN17338_c0_g4	PREDICTED: F-box/kelch-repeat protein At3g06240
A	MEcyan	TRINITY_DN54030_c0_g1	-
A	MEcyan	TRINITY_DN4330_c0_g3	PREDICTED: metacaspase-9-like
A	MEcyan	TRINITY_DN5081_c0_g3	UPF0202 plant-like protein, partial
A	MEcyan	TRINITY_DN1909_c0_g1	-
A	MEcyan	TRINITY_DN10911_c0_g1	-
A	MEcyan	TRINITY_DN35356_c0_g1	unknown
A	MEcyan	TRINITY_DN19797_c0_g1	-
A	MEcyan	TRINITY_DN12520_c0_g1	-
A	MEcyan	TRINITY_DN1732_c0_g1	LEAF RUST 10 DISEASE-RESISTANCE LOCUS RECEPTOR-LIKE PROTEIN KINASE-like 2.1
A	MEcyan	TRINITY_DN4330_c0_g2	metacaspase-9-like
A	MEcyan	TRINITY_DN9464_c0_g1	Retrovirus-related Pol polyprotein from transposon TNT 1-94
A	MEcyan	TRINITY_DN18774_c0_g1	-
A	MEcyan	TRINITY_DN53137_c0_g1	Retrovirus-related Pol polyprotein from transposon TNT 1-94
A	MEcyan	TRINITY_DN1650_c1_g1	PREDICTED: berberine bridge enzyme-like 15
A	MEred	TRINITY_DN117613_c0_g1	PREDICTED: Chenopodium quinoa histone H2B (LOC110684102), mRNA
A	MEred	TRINITY_DN12627_c0_g1	putative Zinc finger, BED-type
A	MEred	TRINITY_DN72273_c0_g1	-
A	MEred	TRINITY_DN14909_c0_g2	PREDICTED: Nicotiana tomentosiformis pentatricopeptide repeat-containing protein At5g01110-like (LOC104120724), mRNA
A	MEred	TRINITY_DN19916_c0_g1	two-component response regulator ARR17-like isoform X2
A	MEred	TRINITY_DN76812_c1_g1	E3 ubiquitin-protein ligase WAV3-like isoform X1
A	MEred	TRINITY_DN25780_c0_g1	-
A	MEred	TRINITY_DN13429_c0_g1	peroxidase 57-like
A	MEred	TRINITY_DN10172_c0_g1	protein INVOLVED IN DE NOVO 2-like
A	MEred	TRINITY_DN17973_c0_g1	-
A	MEred	TRINITY_DN16879_c0_g3	HDEM genome, scaffold: C4
A	MEred	TRINITY_DN24091_c0_g1	unnamed protein product
A	MEred	TRINITY_DN16266_c1_g2	-
A	MEred	TRINITY_DN12436_c0_g1	B3 domain-containing protein Os01g0723500-like
A	MEred	TRINITY_DN33135_c0_g1	putative laccase
A	MEred	TRINITY_DN604_c0_g3	-
A	MEred	TRINITY_DN11747_c1_g1	germin-like protein subfamily 1 member 15
A	MEred	TRINITY_DN54_c0_g1	-
A	MEred	TRINITY_DN28867_c0_g1	-
A	MEred	TRINITY_DN42938_c0_g1	-
A	MEred	TRINITY_DN10309_c0_g1	-
A	MEred	TRINITY_DN18453_c0_g1	-
A	MEred	TRINITY_DN5678_c0_g1	-
A	MEred	TRINITY_DN108429_c0_g2	jasmonate-induced protein homolog
A	MEred	TRINITY_DN26096_c0_g1	-
A	MEred	TRINITY_DN18405_c0_g1	S-adenosylmethionine synthase 2-like

A	MEred	TRINITY_DN56410_c0_g1	-
A	MEred	TRINITY_DN11577_c1_g1	Transposon TX1 uncharacterized 149 kDa protein
A	MEred	TRINITY_DN2287_c0_g2	glutaminyl-peptide cyclotransferase
A	MEred	TRINITY_DN14293_c0_g1	-
A	MEred	TRINITY_DN69332_c0_g1	xanthoxin dehydrogenase-like
A	MEred	TRINITY_DN9405_c0_g1	-
B	MEorange	TRINITY_DN12766_c0_g1	-
B	MEorange	TRINITY_DN265_c0_g2	zinc finger BED domain-containing protein RICESLEEPER 1-like
B	MEorange	TRINITY_DN2442_c1_g2	PREDICTED: jasmonate-induced protein homolog
B	MEorange	TRINITY_DN33728_c0_g1	-
B	MEorange	TRINITY_DN34272_c0_g1	-
B	MEorange	TRINITY_DN3427_c1_g1	Ubiquitin-conjugating enzyme
B	MEorange	TRINITY_DN5000_c0_g2	-
B	MEorange	TRINITY_DN620_c0_g1	PREDICTED: plant UBX domain-containing protein 7
B	MEorange	TRINITY_DN773_c0_g3	PREDICTED: jasmonate-induced protein homolog
B	MEorange	TRINITY_DN7824_c0_g1	vacuolar protein sorting 34
B	MEorange	TRINITY_DN5649_c0_g1	calmodulin-binding family protein
B	MEorange	TRINITY_DN20832_c0_g1	kunitz trypsin inhibitor 2-like
B	MEorange	TRINITY_DN62788_c0_g1	proteinase inhibitor I3
B	MEorange	TRINITY_DN60890_c1_g1	ribonuclease H protein
B	MEorange	TRINITY_DN888_c0_g1	LOW QUALITY PROTEIN: branched-chain-amino-acid aminotransferase-like protein 1
B	MEorange	TRINITY_DN9338_c0_g1	ricin B-like lectin R40G3
B	MEorange	TRINITY_DN18389_c0_g1	-
B	MEorange	TRINITY_DN35108_c0_g1	PREDICTED: probable glycosyltransferase At5g03795 isoform X2
B	MEorange	TRINITY_DN15137_c0_g1	zinc finger BED domain-containing protein DAYSLEEPER-like
B	MEorange	TRINITY_DN2286_c4_g2	-
B	MEorange	TRINITY_DN3738_c0_g2	-
B	MEorange	TRINITY_DN3303_c0_g1	-
B	MEorange	TRINITY_DN50087_c0_g1	folate synthesis bifunctional protein, mitochondrial-like
B	MEorange	TRINITY_DN722_c0_g1	-
B	MEorange	TRINITY_DN7947_c1_g1	PREDICTED: transcription initiation factor TFIID subunit 15b
B	MEorange	TRINITY_DN1407_c0_g1	PREDICTED: vacuolar protein sorting-associated protein 53 A
B	MEsaddlebrown	TRINITY_DN27754_c1_g1	40S ribosomal protein S29
B	MEsaddlebrown	TRINITY_DN671_c0_g2	glutathione S-transferase T1
B	MEsaddlebrown	TRINITY_DN10507_c0_g1	uricase-2 isozyme 2-like
B	MEsaddlebrown	TRINITY_DN9306_c0_g2	-
B	MEsaddlebrown	TRINITY_DN1854_c0_g2	-
B	MEsaddlebrown	TRINITY_DN4213_c0_g1	PREDICTED: transcription initiation factor TFIID subunit 15
B	MEsaddlebrown	TRINITY_DN1155_c0_g1	sphingosine kinase 1-like isoform X1
B	MEsaddlebrown	TRINITY_DN14333_c0_g1	PREDICTED: DExH-box ATP-dependent RNA helicase DExH3
B	MEsaddlebrown	TRINITY_DN604_c1_g1	-
B	MEsaddlebrown	TRINITY_DN5556_c0_g1	PREDICTED: zinc finger CCCH domain-containing protein 15 homolog
B	MEsaddlebrown	TRINITY_DN7811_c0_g1	plant invertase/pectin methylesterase inhibitor
B	MEsaddlebrown	TRINITY_DN1657_c0_g1	cinnamoyl-CoA reductase 1-like isoform X2
B	MEsaddlebrown	TRINITY_DN65103_c0_g1	lectin-like
B	MEsaddlebrown	TRINITY_DN89_c0_g1	-
B	MEsaddlebrown	TRINITY_DN22193_c0_g1	-
B	MEsaddlebrown	TRINITY_DN33659_c1_g1	PREDICTED: DUF21 domain-containing protein At1g47330

B	MEsaddlebrown	TRINITY_DN7316_c0_g1	mitochondrial outer membrane protein porin 4-like
B	MEsaddlebrown	TRINITY_DN5342_c0_g1	PREDICTED: B-cell receptor-associated protein 31-like
B	MEsaddlebrown	TRINITY_DN5401_c0_g1	putative wall-associated receptor kinase-like 16
B	MEsaddlebrown	TRINITY_DN34403_c0_g2	DNA-directed RNA polymerase III subunit 2-like, partial
B	MEsaddlebrown	TRINITY_DN74245_c0_g1	-
B	MEsaddlebrown	TRINITY_DN5928_c0_g1	-
B	MEsaddlebrown	TRINITY_DN1169_c0_g1	-
B	MEsaddlebrown	TRINITY_DN1153_c0_g1	-
B	MEsaddlebrown	TRINITY_DN9536_c0_g1	casein kinase 1-like protein 10 isoform X1
B	MEsaddlebrown	TRINITY_DN1900_c0_g1	PREDICTED: protein FAR1-RELATED SEQUENCE 5-like
B	MEsaddlebrown	TRINITY_DN58932_c0_g1	receptor homology region, transmembrane domain- and RING domain-containing protein 2-like
B	MEsaddlebrown	TRINITY_DN793_c1_g1	PREDICTED: bromodomain-containing protein DDB_G0270170
B	MEsaddlebrown	TRINITY_DN443_c3_g1	-
B	MEsaddlebrown	TRINITY_DN16569_c0_g3	-
B	MEyellowgreen	TRINITY_DN76498_c0_g1	-
B	MEyellowgreen	TRINITY_DN254_c3_g1	-
B	MEyellowgreen	TRINITY_DN13855_c0_g1	-
B	MEyellowgreen	TRINITY_DN23150_c0_g2	-
B	MEyellowgreen	TRINITY_DN94488_c0_g1	PREDICTED: RNA-directed DNA polymerase homolog
B	MEyellowgreen	TRINITY_DN9035_c0_g1	-
B	MEyellowgreen	TRINITY_DN13287_c0_g1	-
B	MEyellowgreen	TRINITY_DN14931_c0_g1	-
B	MEyellowgreen	TRINITY_DN77555_c0_g1	-
B	MEyellowgreen	TRINITY_DN52920_c0_g1	-
B	MEyellowgreen	TRINITY_DN254_c0_g1	-
B	MEyellowgreen	TRINITY_DN4574_c1_g1	-
B	MEyellowgreen	TRINITY_DN136263_c0_g1	-
B	MEyellowgreen	TRINITY_DN16274_c0_g1	-
B	MEyellowgreen	TRINITY_DN6814_c0_g1	transposable element gene
B	MEyellowgreen	TRINITY_DN254_c12_g1	-
B	MEyellowgreen	TRINITY_DN9550_c0_g2	-
B	MEyellowgreen	TRINITY_DN36502_c0_g1	-
B	MEyellowgreen	TRINITY_DN29675_c0_g1	-
B	MEyellowgreen	TRINITY_DN25898_c0_g1	-
B	MEyellowgreen	TRINITY_DN59546_c0_g1	-
B	MEyellowgreen	TRINITY_DN14603_c0_g2	putative LRR receptor-like serine/threonine-protein kinase
B	MEyellowgreen	TRINITY_DN12145_c0_g2	-
B	MEyellowgreen	TRINITY_DN14753_c0_g2	-
B	MEyellowgreen	TRINITY_DN254_c4_g1	-
B	MEyellowgreen	TRINITY_DN254_c13_g1	-
B	MEyellowgreen	TRINITY_DN23695_c0_g3	unnamed protein product
B	MEyellowgreen	TRINITY_DN43438_c0_g2	-
B	MEyellowgreen	TRINITY_DN24271_c0_g1	-
B	MEyellowgreen	TRINITY_DN125242_c0_g1	-
B	MEyellowgreen	TRINITY_DN29182_c1_g1	-
B	MEyellowgreen	TRINITY_DN254_c9_g2	-
B	MEyellowgreen	TRINITY_DN60817_c0_g1	-
B	MEyellowgreen	TRINITY_DN48694_c0_g1	-
B	MEyellowgreen	TRINITY_DN4062_c0_g1	-

B	MEyellowgreen	TRINITY_DN35143_c0_g1	-
B	MEyellowgreen	TRINITY_DN34826_c0_g1	-
B	MEyellowgreen	TRINITY_DN11764_c0_g1	transposable element gene
B	MEyellowgreen	TRINITY_DN254_c5_g1	-
B	MEyellowgreen	TRINITY_DN2861_c0_g1	-
B	MEyellowgreen	TRINITY_DN50309_c0_g1	-
B	MEyellowgreen	TRINITY_DN131418_c0_g1	-
L	MEdarkolivegreen	TRINITY_DN856_c0_g2	-
L	MEdarkolivegreen	TRINITY_DN986_c0_g3	-
L	MEdarkolivegreen	TRINITY_DN69550_c0_g2	-
L	MEdarkolivegreen	TRINITY_DN53545_c0_g1	elongation factor 1-alpha-like
L	MEdarkolivegreen	TRINITY_DN1597_c0_g2	synaptosomal-associated protein 25-like
L	MEdarkolivegreen	TRINITY_DN147359_c0_g1	cytochrome c oxidase subunit II
L	MEdarkolivegreen	TRINITY_DN18894_c0_g2	actin, clone 403
L	MEdarkolivegreen	TRINITY_DN20883_c0_g1	cytochrome oxidase subunit 3
L	MEdarkolivegreen	TRINITY_DN149768_c0_g1	cytochrome c oxidase subunit I
L	MEdarkolivegreen	TRINITY_DN14499_c0_g1	-
L	MEdarkolivegreen	TRINITY_DN57790_c0_g1	-
L	MEdarkolivegreen	TRINITY_DN25648_c0_g1	-
L	MEdarkolivegreen	TRINITY_DN520_c1_g1	-
L	MEdarkolivegreen	TRINITY_DN41658_c0_g1	-
L	MEdarkolivegreen	TRINITY_DN56356_c1_g1	-
L	MEdarkolivegreen	TRINITY_DN69910_c0_g1	PREDICTED: cytochrome P450 82G1
L	MEdarkolivegreen	TRINITY_DN45308_c0_g1	PREDICTED: carboxyl-terminal-processing peptidase 3, chloroplastic
L	MEdarkolivegreen	TRINITY_DN3009_c2_g1	disease resistance protein RGA2-like
L	MEdarkolivegreen	TRINITY_DN17195_c0_g1	Retrovirus-related Pol polyprotein from transposon TNT 1-94
L	MEdarkolivegreen	TRINITY_DN2424_c0_g1	zinc finger BED domain-containing protein RICESLEEPER 2-like
L	MEdarkolivegreen	TRINITY_DN95257_c0_g1	PREDICTED: putative disease resistance protein RGA1
L	MEdarkolivegreen	TRINITY_DN27593_c0_g1	Protein NRT1/ PTR family 8.3
L	MEdarkolivegreen	TRINITY_DN19067_c0_g1	putative LOV domain-containing protein
L	MEdarkolivegreen	TRINITY_DN91038_c0_g1	-
L	MEdarkolivegreen	TRINITY_DN17872_c4_g1	putative ribonuclease H protein
L	MEdarkolivegreen	TRINITY_DN6717_c0_g2	tropinone reductase homolog At5g06060-like
L	MEsteelblue	TRINITY_DN16399_c0_g1	protein TIFY 10A-like
L	MEsteelblue	TRINITY_DN19776_c0_g1	PREDICTED: guanylate kinase 1
L	MEsteelblue	TRINITY_DN25389_c0_g1	serine/threonine-protein kinase ATG1a-like
L	MEsteelblue	TRINITY_DN4496_c0_g1	PREDICTED: protein TIFY 10A-like
L	MEsteelblue	TRINITY_DN38203_c0_g1	-
L	MEsteelblue	TRINITY_DN16609_c0_g1	dCTP pyrophosphatase 1-like isoform X1
L	MEsteelblue	TRINITY_DN4648_c1_g1	PREDICTED: glutamate receptor 3.3 isoform X1
L	MEsteelblue	TRINITY_DN14340_c0_g1	plastidic glucose 6-phoaphate/phosphate translocator2
L	MEsteelblue	TRINITY_DN9048_c0_g1	-
L	MEsteelblue	TRINITY_DN26629_c0_g1	uncharacterized acetyltransferase At3g50280-like
L	MEsteelblue	TRINITY_DN3106_c0_g1	-
L	MEsteelblue	TRINITY_DN1078_c0_g1	peroxidase, partial
L	MEsteelblue	TRINITY_DN47636_c0_g1	protein TIFY 10B-like
L	MEsteelblue	TRINITY_DN5765_c0_g2	uncharacterized membrane protein At4g09580-like
L	MEsteelblue	TRINITY_DN7520_c0_g1	vacuolar-sorting receptor 4-like
L	MEsteelblue	TRINITY_DN8395_c0_g1	PREDICTED: serine carboxypeptidase-like

L	MEsteelblue	TRINITY_DN16696_c0_g1	-
L	MEsteelblue	TRINITY_DN4137_c0_g1	probable protein phosphatase 2C 33
L	MEsteelblue	TRINITY_DN10927_c0_g1	transmembrane 9 superfamily member 3
L	MEsteelblue	TRINITY_DN17599_c0_g1	allene oxide synthase 2-like isoform X2
L	MEsteelblue	TRINITY_DN50935_c0_g1	O-acetyltransferase family protein isoform 3
L	MEsteelblue	TRINITY_DN7695_c0_g1	PREDICTED: tryptophan synthase beta chain 1-like isoform X2
L	MEsteelblue	TRINITY_DN89324_c0_g1	Eukaryotic initiation factor 4A-9
L	MEsteelblue	TRINITY_DN2028_c18_g1	-
L	MEsteelblue	TRINITY_DN24234_c0_g1	1-deoxyxylulose 5-phosphate synthase
L	MEsteelblue	TRINITY_DN18944_c0_g2	protein TIFY 5A-like
L	MEsteelblue	TRINITY_DN10166_c0_g1	auxin response factor 9-like
L	MEsteelblue	TRINITY_DN45186_c0_g1	-
L	MEsteelblue	TRINITY_DN5074_c0_g1	annexin D3-like isoform X2
L	MEsteelblue	TRINITY_DN4950_c1_g2	-
L	MEsteelblue	TRINITY_DN12557_c0_g1	linoleate 13S-lipoxygenase 2-1, chloroplastic-like
L	MEsteelblue	TRINITY_DN13556_c0_g2	PREDICTED: hydroquinone glucosyltransferase
L	MEsteelblue	TRINITY_DN8272_c0_g1	putative glycosyltransferase 7
L	MEsteelblue	TRINITY_DN1417_c1_g1	-
L	MEsteelblue	TRINITY_DN1783_c0_g1	polygalacturonase inhibitor 1
L	MEsteelblue	TRINITY_DN2391_c1_g2	1-aminocyclopropane-1-carboxylate oxidase homolog 4-like

Supplementary Table 6. Selected DEGs candidates and Hub genes in each top WGCNA module correlated with bract color L-a-b values.

Lab	DEGs or module genes	Gene ID	Gene name	Name in Figure 7	NR annotation
A	MEdarkgrey	TRINITY_DN12271_c0_g2	*SGR*	*SGR* (1)	protein STAY-GREEN 1, chloroplastic-like
A	MEdarkgrey	TRINITY_DN5726_c0_g1	*RCHY1*	*RCHY1*	E3 ubiquitin-protein ligase MIEL1-like isoform X2
A	MEdarkgrey	TRINITY_DN19569_c0_g1	*GERD*	*GERD* (1)	(-)-germacrene D synthase-like
A	MEdarkgrey	TRINITY_DN9344_c0_g1	*SGR*	*SGR*	protein STAY-GREEN 1, chloroplastic-like
A	MEred	TRINITY_DN18405_c0_g1	*metK*	*metK* (1)	S-adenosylmethionine synthase 2-like
A	MEred	TRINITY_DN69332_c0_g1	*ABA2*	*ABA2* (1)	xanthoxin dehydrogenase-like
A	MEred	TRINITY_DN9405_c0_g1	*ABA2*	*ABA2* (2)	xanthoxin dehydrogenase
A	MEred	TRINITY_DN24091_c0_g1	*NOL*	*NOL*	chlorophyll b reductase
A	MEred	TRINITY_DN5678_c0_g1	*DHDDS*	*DHDDS*	ditrans,polycis-polyprenyl diphosphate synthase
A	MEcyan	TRINITY_DN47558_c2_g1	*UBE2A*	*UBE2A*	ubiquitin-conjugating enzyme E2 A
A	MEcyan	TRINITY_DN5772_c0_g1	*AOC*	*AOC*	allene oxide cyclase, chloroplastic-like
A	MEcyan	TRINITY_DN112782_c0_g1	*HSP90A*	*HSP90A*	molecular chaperone HtpG
A	MEcyan	TRINITY_DN1650_c1_g1	*K22395*	*K22395*	cinnamyl-alcohol dehydrogenase
A	DEGs candidates	TRINITY_DN8975_c1_g1	*E1.11.1.7*	*E1.11.1.7*	peroxidase 42
A	DEGs candidates	TRINITY_DN19569_c0_g1	*GERD*	*GERD* (2)	(-)-germacrene D synthase-like
A	DEGs candidates	TRINITY_DN134954_c0_g1	*CHS*	*CHS*	chalcone synthase
A	DEGs candidates	TRINITY_DN34428_c0_g1	*metK*	*metK* (2)	S-adenosylmethionine synthase 2-like
B	MEyellowgreen	TRINITY_DN29182_c1_g1	*APEH*	*APEH*	acylaminoacyl-peptidase
B	MEyellowgreen	TRINITY_DN50309_c0_g1	*SPPL2B*	*SPPL2B*	signal peptide peptidase-like 2B
B	MEyellowgreen	TRINITY_DN254_c3_g1	*TFC3*	*TFC3*	transcription factor C subunit 3
B	MEyellowgreen	TRINITY_DN16274_c0_g1	*PLC*	*PLC*	phospholipase C
B	MEorange	TRINITY_DN34272_c0_g1	*ABCA3*	*ABCA3*	ATP-binding cassette, subfamily A (ABC1), member 3
B	MEorange	TRINITY_DN620_c0_g1	*UBXN7*	*UBXN7*	plant UBX domain-containing protein 7
B	MEorange	TRINITY_DN7824_c0_g1	*PIK3C3*	*PIK3C3*	vacuolar protein sorting 34
B	MEorange	TRINITY_DN888_c0_g1	*EEF2KMT*	*EEF2KMT*	protein-lysine N-methyltransferase
B	MEorange	TRINITY_DN3303_c0_g1	*CARM1*	*CARM1*	type I protein arginine methyltransferase
B	MEorange	TRINITY_DN9914_c0_g1	*FG3*	*FG3*	UDP-glycosyltransferase 79B30-like
B	MEsaddlebrown	TRINITY_DN27754_c1_g1	*RP-S29e*	*RP-S29e*	40S ribosomal protein S29
B	MEsaddlebrown	TRINITY_DN671_c0_g2	*GST*	*GST*	glutathione S-transferase T1
B	MEsaddlebrown	TRINITY_DN10507_c0_g1	*uaZ*	*uaZ*	uricase-2 isozyme 2-like
B	MEsaddlebrown	TRINITY_DN1155_c0_g1	*SPHK*	*SPHK*	sphingosine kinase 1-like isoform X1
B	MEsaddlebrown	TRINITY_DN14333_c0_g1	*DHX36*	*DHX36*	DExH-box ATP-dependent RNA helicase DExH3
B	DEGs candidates	TRINITY_DN6496_c0_g2	*AOC3*	*AOC3*	primary-amine oxidase
B	DEGs candidates	TRINITY_DN24158_c0_g1	*CYP716A*	*CYP716A*	cytochrome P450
B	DEGs candidates	TRINITY_DN41301_c0_g1	*CYP76C*	*CYP76C*	cytochrome P450 76T24-like
L	MESteelblue	TRINITY_DN16399_c0_g1	*JAZ*	*JAZ* (1)	jasmonate ZIM domain-containing protein
L	MESteelblue	TRINITY_DN4496_c0_g1	*JAZ*	*JAZ* (2)	jasmonate ZIM domain-containing protein
L	MESteelblue	TRINITY_DN38203_c0_g1	*JAZ*	*JAZ* (3)	jasmonate ZIM domain-containing protein
L	MESteelblue	TRINITY_DN47636_c0_g1	*JAZ*	*JAZ* (4)	jasmonate ZIM domain-containing protein
L	MESteelblue	TRINITY_DN7695_c0_g1	*trpB*	*trpB*	PREDICTED: tryptophan synthase beta chain 1-like isoform X2
L	MESteelblue	TRINITY_DN16609_c0_g1	*DCTPP1*	*DCTPP1*	dCTP pyrophosphatase 1-like isoform X1
L	MEdarkolivegreen	TRINITY_DN147359_c0_g1	*COX2*	*COX2*	cytochrome c oxidase subunit II
L	MEdarkolivegreen	TRINITY_DN20883_c0_g1	*COX3*	*COX3*	cytochrome oxidase subunit 3
L	MEdarkolivegreen	TRINITY_DN149768_c0_g1	*COX1*	*COX1*	cytochrome c oxidase subunit I
L	MEdarkolivegreen	TRINITY_DN69910_c0_g1	*CYP82G1*	*CYP82G1*	cytochrome P450 82G1
L	MEdarkolivegreen	TRINITY_DN14499_c0_g1	*ATPeF0A*	*ATPeF0A*	F-type H+-transporting ATPase subunit a
L	DEGs candidates	TRINITY_DN1078_c0_g1	*E1.11.1.7*	*E1.11.1.7*	peroxidase, partial

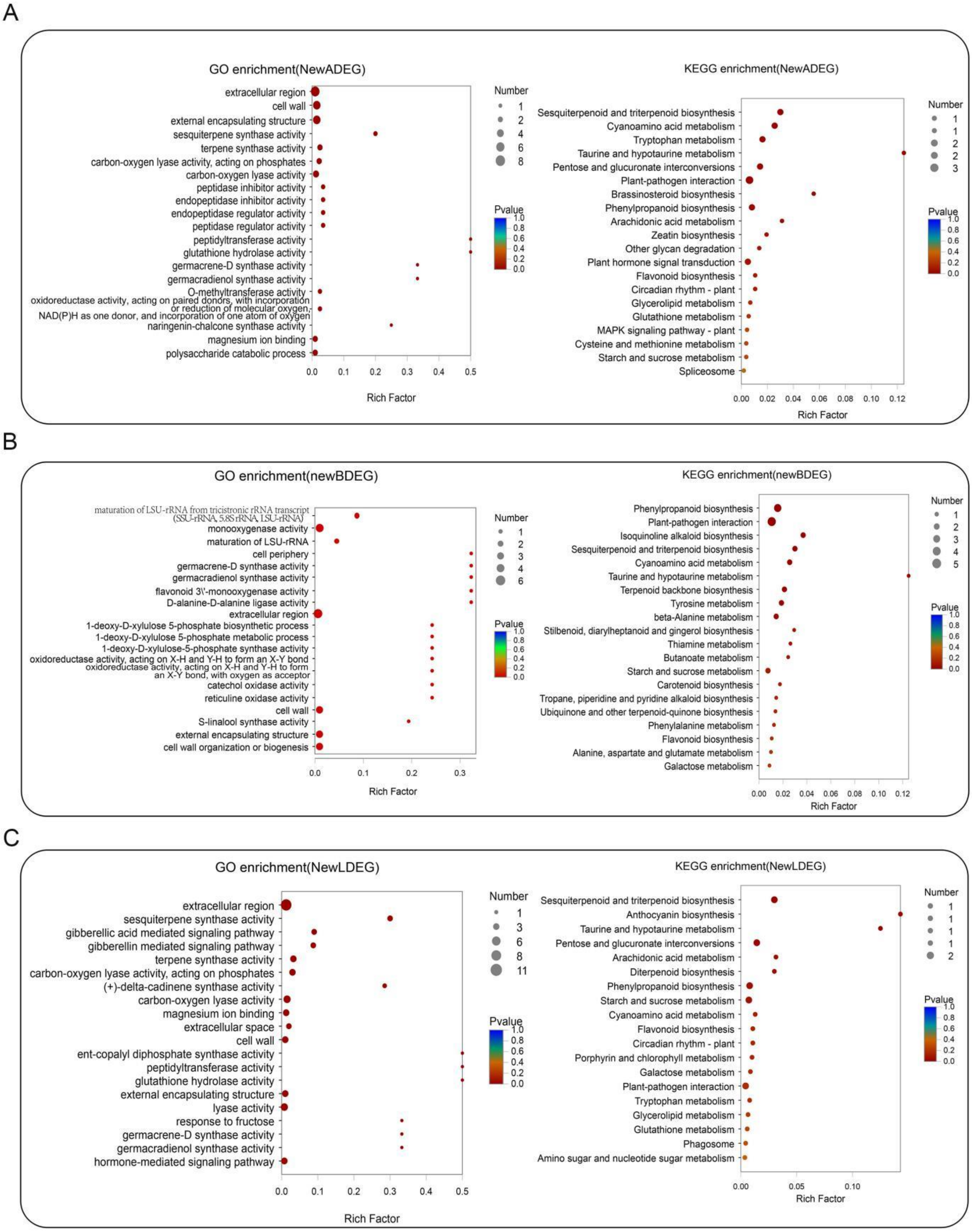

Supplementary Figure 1. GO and KEGG enrichments for genes from DEG analysis. GO (left) and KEGG (right) enrichments for genes based on the pairwise contrast comparisons of bract color Aa **(A)**, Ab **(B)** and AL **(C)** values. The color bar means the *P*-adjust value, while the circle shows the number of enriched genes.
